# Arginyltransferase knockdown attenuates cardiac hypertrophy and fibrosis through TAK1-JNK1/2 pathway

**DOI:** 10.1038/s41598-019-57379-7

**Published:** 2020-01-17

**Authors:** Kanika Singh, Ankit Gupta, Ashish Sarkar, Ishita Gupta, Santanu Rana, Sagartirtha Sarkar, Sameena Khan

**Affiliations:** 1 Drug Discovery Research Centre, Translational Health Science and Technology Institute, Faridabad, Haryana India; 2 Department of Zoology, University of Calcutta, Kolkata, India; 3 Structural Immunology Group, International Centre for Genetic Engineering and Biotechnology, New Delhi, Delhi India

**Keywords:** Cell biology, Cardiology

## Abstract

Myocardial hypertrophy, an inflammatory condition of cardiac muscles is a maladaptive response of the heart to biomechanical stress, hemodynamic or neurohormonal stimuli. Previous studies indicated that knockout of Arginyltransferase (ATE1) gene in mice and embryos leads to contractile dysfunction, defective cardiovascular development, and impaired angiogenesis. Here we found that in adult rat model, downregulation of ATE1 mitigates cardiac hypertrophic, cardiac fibrosis as well as apoptosis responses in the presence of cardiac stress i.e. renal artery ligation. On contrary, in wild type cells responding to renal artery ligation, there is an increase of cellular ATE1 protein level. Further, we have shown the cardioprotective role of ATE1 silencing is mediated by the interruption of TAK1 activity-dependent JNK1/2 signaling pathway. We propose that ATE1 knockdown in presence of cardiac stress performs a cardioprotective action and the inhibition of its activity may provide a novel approach for the treatment of cardiac hypertrophy.

## Introduction

Heart failure is a life threatening medical condition, which is increasing in prevalence and is a major cause of morbidity and mortality worldwide^1–3^. One of the important pathophysiological processes contributing to heart failure is the cardiac hypertrophy^4–7^. Hypertrophy is a compensatory response to an increase in pressure or volume overload, where the heart and the cardiomyocytes enlarge to reduce ventricular wall tension and maintain cardiac efficiency and output. However, persistent stress, resulting from a chronic increase in workload or cardiac insults, ultimately result in heart failure^8–10^. Cardiac fibrosis which is indicated by excess synthesis and deposition of collagen and other extracellular matrix proteins is a major participant during hypertrophy, which leads to disruption of organ architecture and leading to heart failure^11–14^. Due to the life threatening impact of heart failure, there is a growing thirst for knowing the novel signaling mechanism, involved in cardiac hypertrophy development. Having an insight into the mechanisms and potential targets implicit in cardiac hypertrophy will provide novel methodologies for the prevention or treatment of cardiovascular disease. Arginyltransferase (ATE1) is an evolutionary conserved enzyme and has a crucial role in multiple biological events^15–17^. The physiological importance of ATE1 in higher eukaryotes has been shown in previous studies where in case of *Mus musculus*, ATE1 deletion contributes to embryonic lethality due to abnormal cardiac morphogenesis and impaired angiogenesis^18^. Later, it was shown that knocking out of ATE1 gene leads to cell autonomous defective cardiac contractility and myofibril development, myocardium integrity and myocyte contractility during embryogenesis^19^. Consequently, suggesting ATE1 is essential for the development of embryonic heart, maturation and the integrity of blood vessels. Furthermore, in case of ATE1 knockout mouse embryos, defective cardiovascular development was observed, due to deregulation in Gαq-PLC/PKC-MEK1-ERK signaling, where the PKC, PLC, and MEK1 activity was attenuated^20^. Interestingly, cardiac myofibril specific knockdown of ATE1 in mice indicated their survival till 6–12 months but eventually, they died after leading to cardiac contractility defects in myofibrils. This suggested that ATE1 has a cell autonomous role and it maintains heart function by regulating myofibril proteins. Its absence in the heart muscles leads to cardiomyocyte specific defects causing progressive heart failure^21,22^. Previous studies explain how ATE1 deletion impacts the phenotype of a normal heart at embryonic stage as well as its cell autonomous role in age related heart defects. Our study focuses on testing the role of ATE1 in adult rat model in the presence of cardiac stress. We found the ATE1 protein levels were increased under pressure induced hypertrophy and downregulation of ATE1 genes under stress leads to reduction in cardiac hypertrophic responses, cardiac fibrosis and cellular apoptosis. Our results support the previous study where the role of ATE1 and arginylation was checked in various models like yeast (*S. cerevisiae strains*: WT BY474 and W303-1A), Mouse embryonic fibroblasts cells and human embryonic kidney cell line (HEK293 T; clone T7) under various types of stress like NaCl, CdCl_2_, H_2_O_2_, high temperature, UV and Staurosporine treatment. It was noticed that promotion of cell death by arginylation depends upon the nature of stressing factor^23^.

Interestingly, distinct roles have been ascribed to the ATE1 enzyme under different conditions. Some studies point to important pro‐apoptotic effects. In drosophila, for example, ATE1 has been shown to degrade Drosophila inhibitor of apoptosis (DIAP) and thereby promote apoptosis^24^. On contrary, apoptosis was induced in response to heat stress in case of ATE1 knockout mouse embryonic fibroblasts whereas wild type MEFs were safe, thereby act as an anti-apoptotic factor^25^. Together all studies suggest that ATE1 gene is indispensable for heart development, maintenance of the normal heart functions and thus has a crucial role in regulating cardiovascular failures. However, role of ATE1 specific to adult cardiomyocytes in presence of hypertrophic stress has still not been explored. To address this question and analyze the specific role of ATE1, we induced cardiac stress through renal artery ligation in adult rats while knocking down the ATE1 gene. Given the above, our present study suggests a crucial role of ATE1 in the heart. Interestingly our data suggests that cardiac specific ATE1 knockdown attenuate cardiac hypertrophic responses both *in vitro* and *in vivo*. Knockdown of ATE1 in the rat heart mitigated the hypertrophic response and noticeably reduced apoptosis and fibrosis. To highlight the importance of molecular mechanism here we demonstrated the effect of ATE1 knockdown on TAK1 phosphorylation, which thereby controls the expression of hypertrophic genes. Phosphorylation of TAK1 is getting diminished in knockdown state upon pressure overload, which then impairs the activation of JNK1/2 mediated hypertrophic pathways. Our finding suggests that deficiency of ATE1 drastically reduced the expression of hypertrophic as well as fibrotic markers and thus plays an important role during cardiac stress.

## Materials and Methods

### Animals and cell line used

24-week-old Wistar rats (Rattus norvegicus) used in this study were procured from NIN, Hyderabad, India. The investigation is in accordance with the guidelines for the Care and Use of Laboratory Animals published by the US National Institute of Health (NIH Publication no. 85-23, revised 1996)^26^. All rats used in this study were maintained in a pathogen-free facility at the University of Calcutta under controlled temperature and humidity conditions. All experiments were conducted in accordance with the National Institutes Health “Guide for the Care and Use of Laboratory Animals” and were approved by the Institutional Animal Ethics Committee, University of Calcutta (Registration no. 885/ac/05/CPCSEA), registered under CPCSEA, MoEF, GOI^26^.

H9C2 rat cardiomyocyte cells were purchased from the American Type Culture Collection (ATCC) and cultured at 37 °C and 5% CO_2_ in Dulbecco’s Modified Eagle Medium (CELL clone) containing 10% Fetal Bovine Serum (Gibco).

### Generation of cardiac hypertrophy *invitro* and *in vivo*

For the generation of *invitro* cardiac hypertrophy model, H9C2 cells were seeded in six-well plates at a density of 0.2 × 10^6^ cells/well. After 24 hours cells were sera starved for 12 hours followed by treatment with Angiotensin II (Ang II) (1 µM) (Sigma Aldrich,4474913) and vehicle alone in control cells for 24 hours. Establishment of hypertrophic responses was determined by fetal gene expression using Real-Time Polymerase Chain Reaction (RT-PCR).

*In vivo*, cardiac hypertrophy model was generated by ligating right renal artery of 28-week-old male rats (250–300 g; *n* = 10), as described before^27^. Rats that underwent a similar procedure without aortic ligation were termed as the sham-operated control group (*n* = 10). Animals were maintained in optimum condition for 14 days and were sacrificed on the 15th day after surgery. Hearts were taken out and stored in liquid nitrogen for future use. Hypertrophy was measured by the heart weight (HW in mg) to body weight (BW in g) ratio^28^.

### ATE1 knockdown *invitro* and *invivo*

H9C2 cells induced with 1 µM Ang II underwent siRNA transfection using DharmaFECT transfection reagent 1 (Dharmacon, T-2001-03) using the manufacturer’s instruction at a final concentration of 25 nM gene specific ATE1 (Dharmacon, L-083990-02-0050) or Non-specific siRNA control (NS) (Dharmacon, D-001810-10-50). The efficiency of knockdown was evaluated using RT-PCR after 24 hours of transfection.

For the generation of ATE1 knockdown hypertrophied rats, ATE1 siRNA was encapsulated in stearic acid-modified carboxy methyl chitosan (CMC) conjugated to a cardiomyocyte-targeting peptide as described previously^29^.ATE1 siRNA (TG) with CMC-peptide was injected intravenously at a dose of 2 mg/kg of body weight into rats in which the renal artery was ligated on alternate days starting from the 8th day until the 14th day of ligation. Rats that underwent a sham operation and treated with nonspecific (NS) siRNA tagged with CMC-peptide were used as negative controls to ATE1 siRNA (Dharmacon)^26^.

### Echocardiography evaluation

Two-dimensional echocardiography was performed to determine cardiac function *in vivo*. Lightly sedated renal artery-ligated and sham-operated rats on the 15th day after surgery were evaluated using the M-mode views on a transthoracic study, measuring left ventricular systolic and diastolic dimensions and fractional shortening (%FS). Data were correlated with the timing of the QRS complex. Digitized images were obtained using an ultrasound system (VividS5 system, GE Healthcare)^27^.

### Total RNA isolation and quantitative Real Time-PCR

Total RNA was extracted from H9C2 cells as well as rat heart samples by using TRIzol reagent (Sigma-Aldrich, T9424). Equal quantities of RNA (1 µg) were converted into cDNA using the oligo (dT) primers with a RevertAid First Strand cDNA synthesis Kit (Thermo Scientific, K1622) according to the manufacturer’s protocol. Quantitative real-time PCR (RT-PCR) amplification of indicated genes was performed using SYBR Green PCR Master Mix (Applied Biosystems,4309155) according to the manufactuer’s instructions. Thermal cycling was done using the 7500 Real Time-PCR System (Applied Biosystems), and the results were analyzed using the 2−ΔΔCT method. The mRNA levels of the target genes were normalized to the values of GAPDH as an internal control. The RT-PCR was carried out using the following primers shown in Supplementary Table S1.

### Protein extraction and western blot analysis

Rat heart samples were homogenized with a homogenizer and lysed using RIPA lysis buffer (50 mM Tris-Cl, pH 8.0, 1% Triton X-100, 10% glycerol, 1 mM EDTA, 250 mM NaCl, 1 mM dithiothreitol and 1 mM phenylmethylsulphonyl fluoride). Protein concentrations were determined using the Quick Start Bradford reagent (Biorad,500-0205). Briefly, 30ug of protein was loaded onto 12% SDS-PAGE electrophoresis gel, and then transferred to polyvinylidene fluoride (PVDF) membranes (Millipore, IPVH00010). After blocking for 1 hour with 5% Bovine serum albumin (Millipore,1.12018.0025) at room temperature and blots were incubated with respective primary antibodies for overnight at 4 °C: anti ANP (novus,NBP2-14873), anti ATE1 (abcam,ab199423), anti ERK1/2 (Cell signaling technology,4695), anti-phospho-ERK1/2 (Cell signaling technology,4377), anti-JNK1/2 (Cell signaling technology,9252), anti-phospho JNK1/2 (Cell signaling technology,4668), GAPDH (Cell signaling technology,5174), anti-TAK1 (Signalway antibody,41477), anti-phospho TAK1 (Signalway antibody,12255), anti SMAD3 (Santa Cruz,sc101154), anti SMAD4 (Santa Cruz,sc7966), Osteopontin (Santa Cruz,sc21742) Caspase3 antibody (Cell signaling technology,9662). Subsequently, the PVDF membranes were incubated with Goat Anti-Rabbit IgG/HRP (Invitrogen,31460) or Goat anti-mouse IgG HRP (Invitrogen,31430) antibody at room temperature for 1 hour. Specific proteins were detected using chemiluminescent HRP substrate (Amersham, RPN2232) and images were obtained by Gel Doc XR + Gel Documentation system (Biorad).

### Caspase activity assay

Following treatment with ATE1 siRNA for 48 hours, H9C2 cells in a 96-black well plate were subjected to 200 nM Ang II treatment for 24 hours followed by caspase activity measurement by Apo-ONE® Homogeneous Caspase-3/7 Assay kit (Promega,G7792). Briefly, the Caspase Substrate and Apo-ONE® Caspase-3/7 Buffer were mixed to make the Apo-ONE® Caspase-3/7 Reagent. 100 μl of this reagent was added to each well, the content of well was gently mixed then incubated at room temperature for 30 minutes. The fluorescence of each sample was measured (499 nm/521 nm) by a Fluorescence Microplate Reader (Synergy HTX).

### Statistical analysis

All data are expressed as the mean ± SE. Comparisons between groups were analyzed by Student’s t-test or ANOVA as appropriate. All statistical analyses were performed with GraphPad Prism software, version 5. *P* values < = 0.05 were considered statistically significant.

## Results

### Generation of cardiac hypertrophy and ATE1 knockdown *in vitro* and *in vivo* model

To interrogate the role of ATE1, H9C2 cell line and a right renal artery ligated rat diseased model was generated to check whether ATE1 has a regulatory role in cardiac hypertrophy that leads towards the fibrosis and apoptosis.

**Figure 1:**
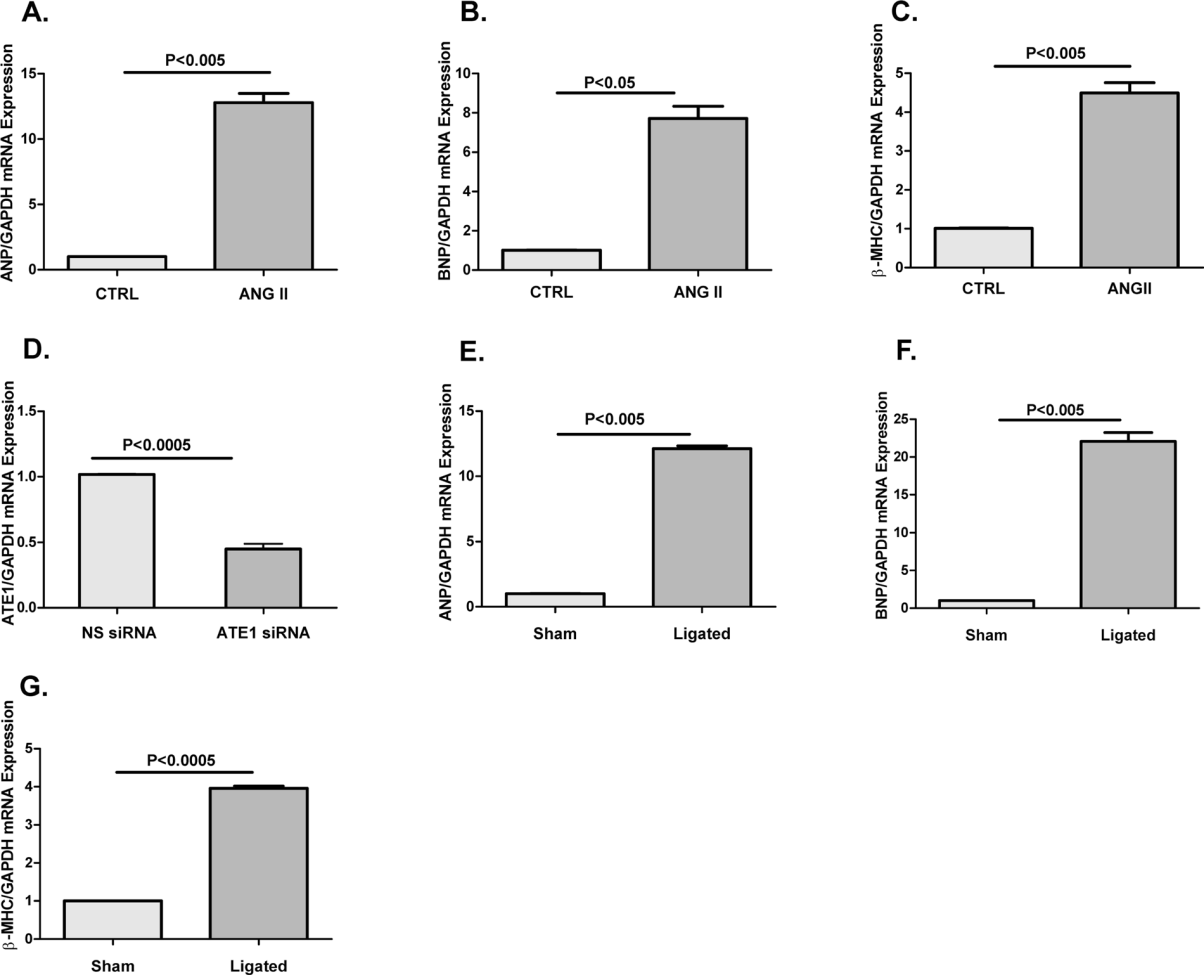
Generation of cardiac hypertrophy and ATE1 knockdown in *invitro* and in *invivo* model Increase in mRNA levels of (**A**) ANP, (**B**) BNP, (**C**) β-MHC in Ang II treated H9C2 cells using Quantitative real-time PCR analysis (**D**) Graph showing significant reduction of ATE1 levels when knockdown by ATE1 siRNA compare to NS siRNA. Quantitative real-time PCR analysis of increased mRNA levels of (**E**) ANP, (**F**) BNP and (**G**) β-MHC in the heart samples of control (Sham) vs Renal artery ligated rat samples (Ligated). Experiment performed in triplicates and normalized to GAPDH content. Statistical analysis is carried out by Student’s two tailed unpaired T test. Data are represented as mean ± SE.

We generated *invitro* hypertrophy in H9C2 cells using Ang II and gene expression of hypertrophy markers were checked in Ang II treated and vehicle treated control (CTRL) cells. Higher mRNA expression of ANP, BNP, and β-MHC indicated the generation of hypertrophic response (Fig. 1A–C). ATE1 knockdown was done in H9C2 cells using ATE1 siRNA, along with which cells were transfected with non-specific control siRNA (NS siRNA). Reduced expression of ATE1 in siRNA treated samples as compared to NS siRNA treated cells indicated a successful knockdown (Fig. 1D). For generating cardiac hypertrophy in an *invivo* rat model, ligation of the right renal artery was done as detailed in the Methods section. We again examined the ANP, BNP and β-MHC expression in the artery ligated (Ligated) and sham operated (Sham) rat sample by Real Time-PCR. Increase in the level of these markers in ligated as compared to the sham indicated the generation of hypertrophy (Fig. 1E–G). Later non-specific siRNA (NS siRNA) and ATE1 siRNA were delivered into renal artery ligated rats which termed as (Ligated + NS siRNA) and (Ligated + ATE1 siRNA) respectively as detailed in the methods section.

### Enhanced ATE1 expression in hypertrophied heart samples

**Figure 2:**
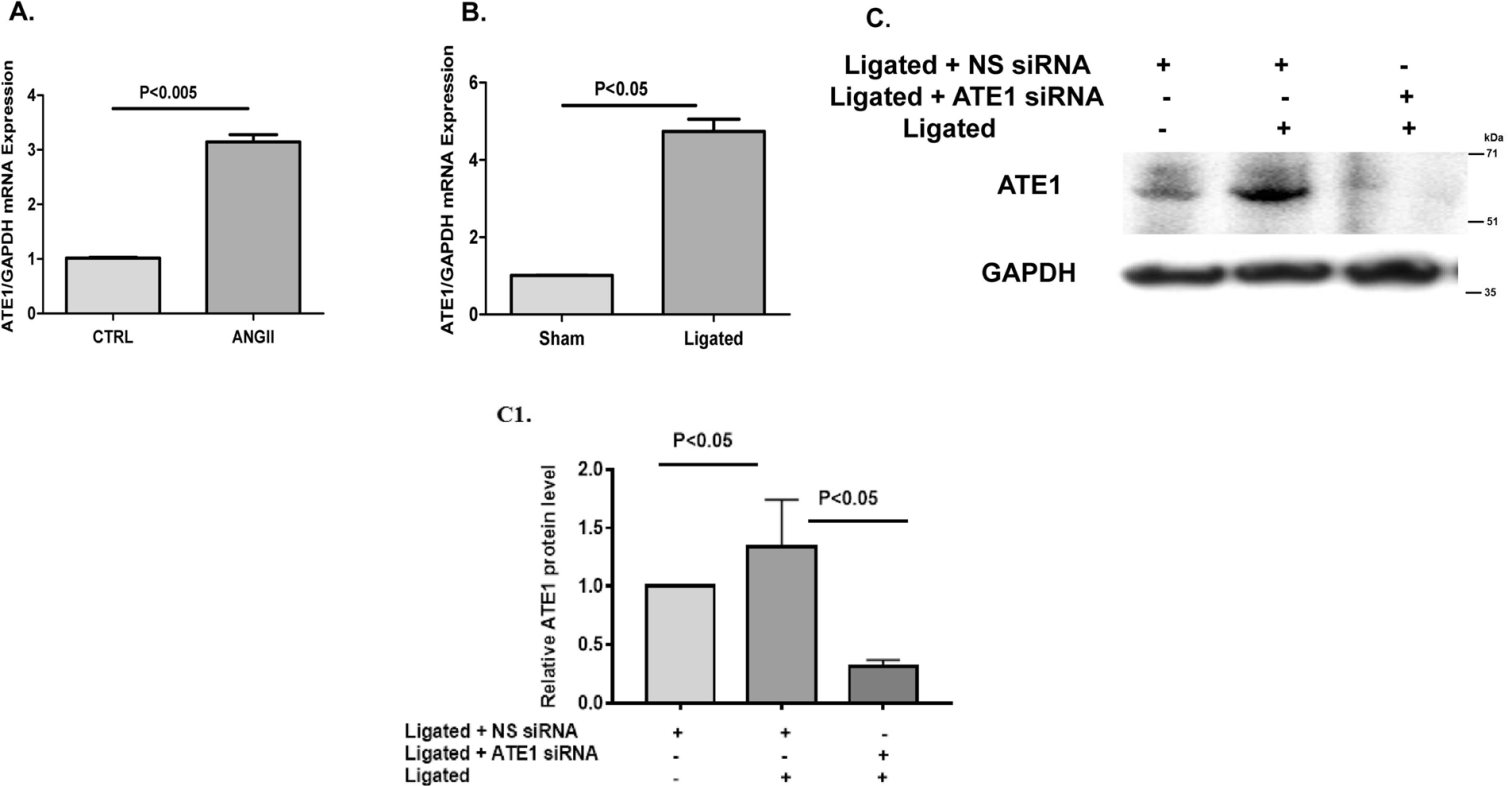
ATE1 expression is upregulated by hypertrophic stimuli. (**A**) Quantitative real-time PCR analysis of mRNA levels of ATE1 in Ang II treated H9C2 cells. (**B**) Transcriptional levels of ATE1 in heart samples from rat subjected to ligation of right renal artery (Ligated) and sham-operated control (Sham) rats. (**C**) Western blot analysis of ATE1 protein levels in heart samples from sham and renal ligated rats. Data were derived from experiments performed in triplicate and normalized to GAPDH content. Statistical analysis was carried out by student’s two-tailed t-test (*shows non-specific binding of antibody).

In order to investigate the probable involvement of ATE1 in the regulation of cardiac hypertrophy, we first explored whether ATE1 expression was changed in angiotensin induced cell-based model as well as an *invivo* rat model of cardiac hypertrophy. Our data showed ATE1 upregulation in H9C2 cells that were stimulated with Ang II when compared with vehicle treated control cells (CTRL) (Fig. 2A). Similarly, enhanced ATE1 mRNA expression was noted in the rat hearts that underwent right renal artery ligation (Ligated) compared with sham-operated control (Sham) (Fig. 2B). Further protein levels in rat samples also confirmed the enhanced ATE1 expression in case of hypertrophic stress (Fig. 2C). Taken together, this increased expression of ATE1 suggests that this gene may be implicated in the development of cardiac hypertrophy.

### Knockdown of ATE1 attenuates cardiac hypertrophy *in vitro* and *in vivo*

**Figure 3:**
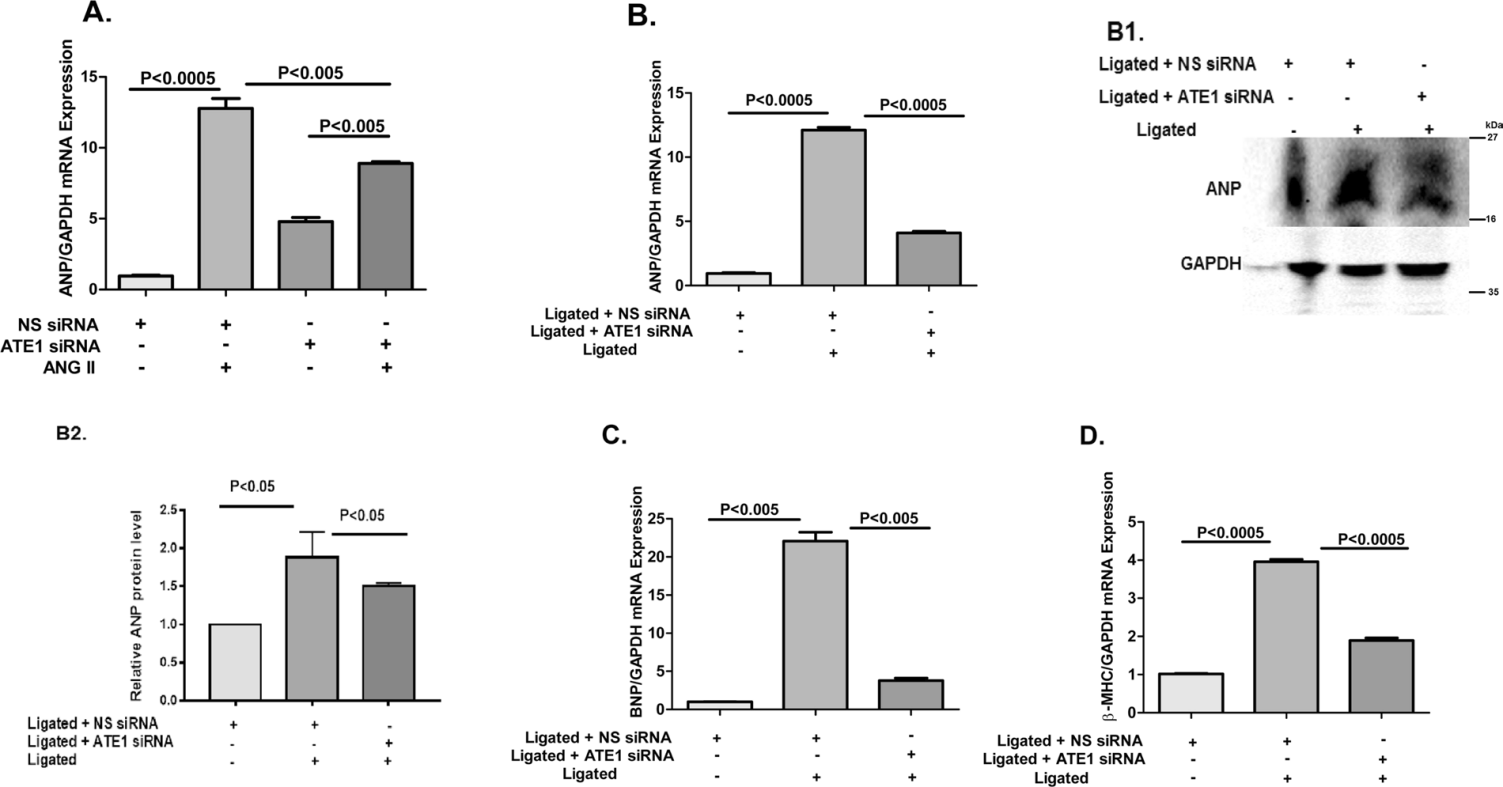
Knockdown of ATE1 attenuates cardiac hypertrophy. Real-time PCR analysis showing the mRNA levels of fetal gene (**A**) ANP in Ang II treated ATE1 knockdown H9C2 cells Decreased mRNA levels in (**B**) ANP (B1) Western blot analysis of ANP protein levels in heart samples from sham and renal ligated rats (B2) bar graph was generated by quantifying the blots and normalizing the intensities of bands to the untreated lane (**C**) BNP and (**D**) β-MHC in Ligated ATE1 siRNA when compared with Ligated NS siRNA samples. Statistical analysis was carried out by one-way ANOVA. Data are represented as mean ± SE.

The increase in ATE1 expression upon hypertrophic stress incited us to investigate whether the knockdown of ATE1 reduces the cardiac hypertrophy. Ang II treated H9C2 cells with ATE1 knockdown notably showed a decreased expression of ANP when compared to the samples treated with Ang II along with non-specific siRNA (Fig. 3A). This data suggests that ATE1 can modulate the hypertrophic growth of myocytes induced by Ang II. We then pursued to validate the above finding in rat cardiac hypertrophied model with cardiac-specific knockdown of ATE1 and found consistency. In response to renal artery ligation, ATE1 knockdown significantly attenuated cardiac hypertrophy (Ligated + ATE1siRNA) as compared where nonspecific siRNA was knockdown (Ligated + NS-siRNA) as shown by the reduced mRNA expression of hypertrophic markers like ANP, BNP, and β-MHC (Fig. 3B–D) and reduced protein expression levels of ANP (Fig. 3B1).

**Figure 4:**
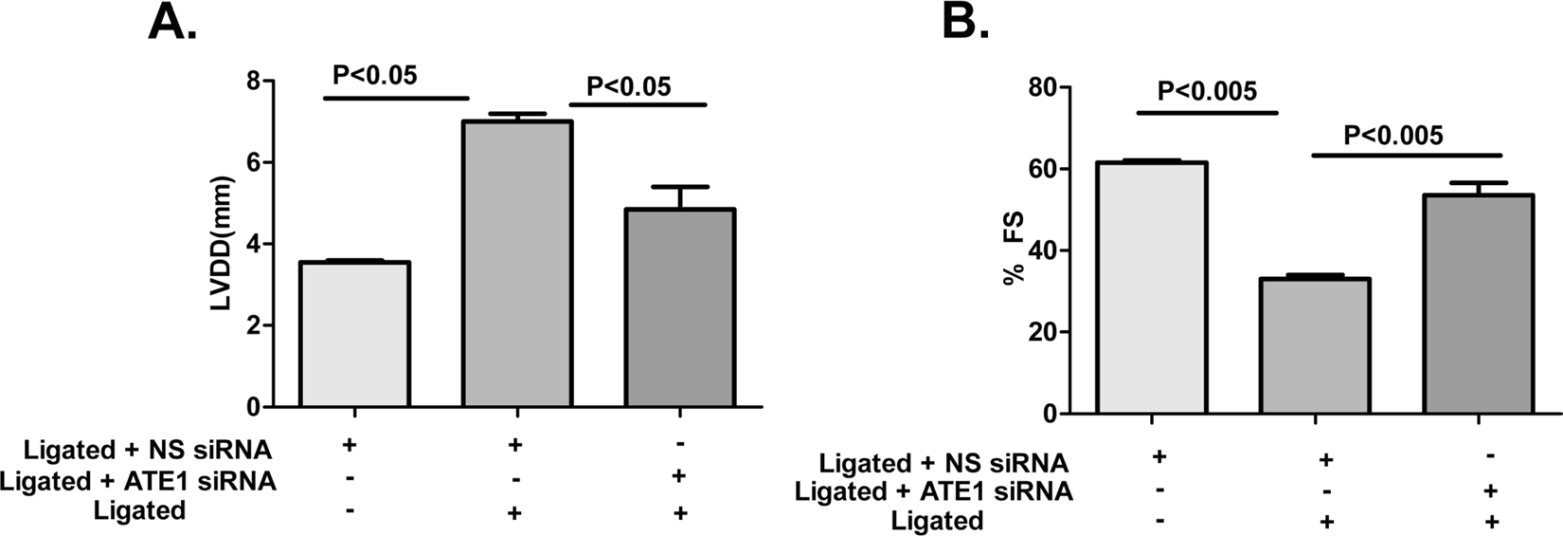
Cardiac ATE1 deficiency restores cardiac dysfunction after right renal artery ligation. Measurements of echocardiographic parameters (**A**) LVDd and (**B**) % FS in the indicated groups.

Furthermore, cardiac function was found to be improved in terms of Left ventricular diastolic dysfunction (LVDd) and Fractional shortening (%FS) in the Ligated + ATE1siRNA as compared to the Ligated + NSsiRNA when examined by M-Mode echocardiography (Supplementary Table S2) (Fig. 4A,B). Echocardiographic measurements confirmed that ATE1 depletion in artery ligated rats prevented ventricular dilation and contractile dysfunction. Broadly, this specifies that the down-regulated ATE1 levels in the heart suppress renal artery ligation-induced cardiac hypertrophic responses and the aftereffect heart dysfunction. Both *in vitro* and *in vivo* experiments surely confirmed a key role of ATE1 during the initiation and progression of pathological cardiac hypertrophy.

### ATE1 knockdown suppresses cardiac hypertrophy depending on TAK1-JNK1/2 signaling activation

To further gain insight into the molecular mechanisms responsible for the effects of ATE1, we investigated the downstream molecular events. We examined the potential effect of ATE1 on TAK1 mediated MAPK signaling pathways, which are well-known pro-hypertrophic kinases cascades comprising three subfamilies: the JNK1/2, ERK1/2, and p38 cascades^30–33^. The MAPK signaling cascade is one of the signaling pathways involved in cardiac hypertrophy^34,35^. After activation downstream p38, JNKs and ERKs each phosphorylate other targets, such as various transcription factors, subsequently leading to reprogramming of cardiac gene expression^36,37^.

**Figure 5:**
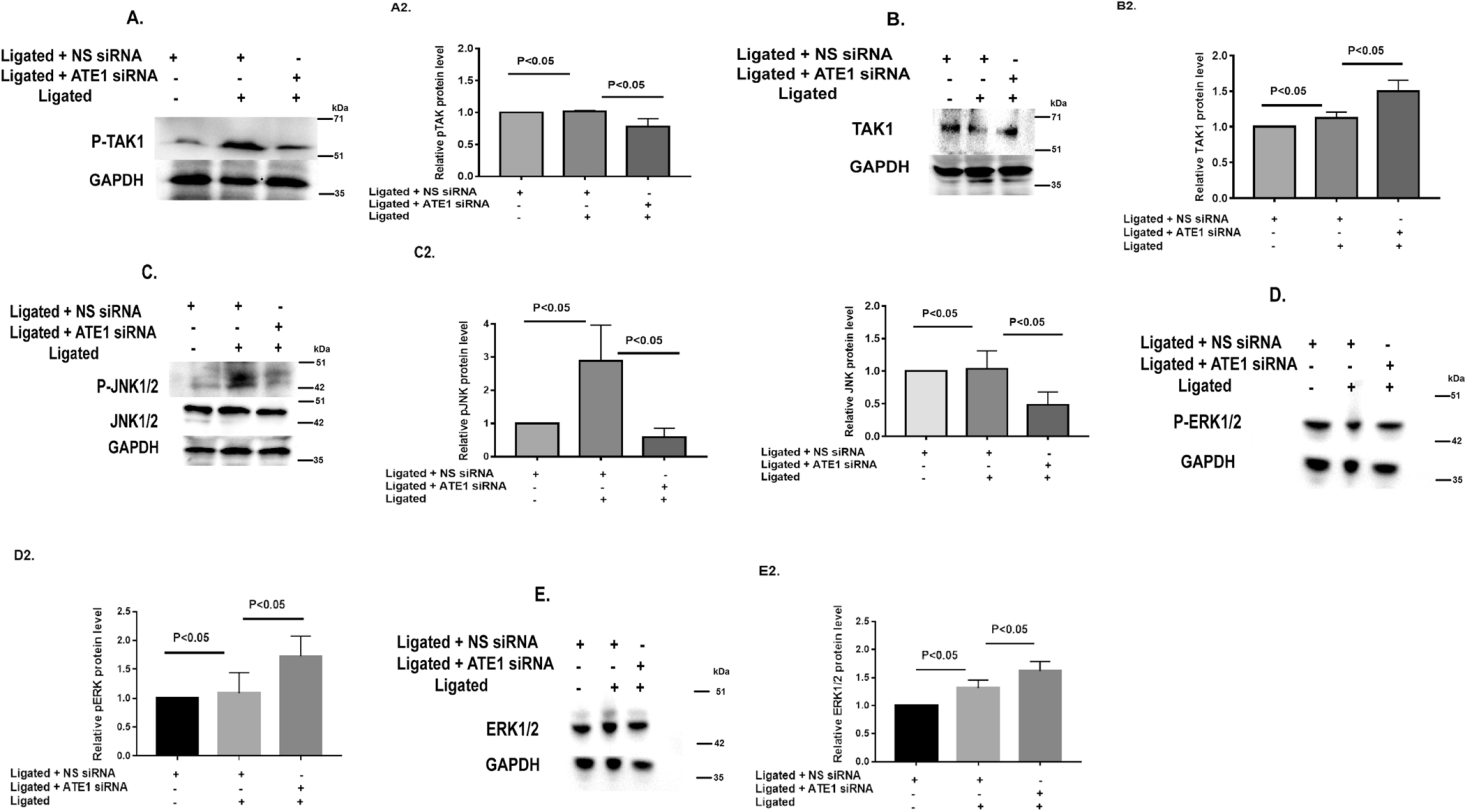
ATE1 knockdown is involved in TAK1-JNK1/2 pathway. Representative western blots and their quantification showing the protein level of (**A**,A2) P-TAK1, (**B**,B2) TAK-1 (**C**,C2) P-JNK1/2 (**D**,D2) P- ERK1/2 (**E**,E2) ERK-1/2 in Ligated ATE1 Si RNA and ligated NS siRNA. Bar graph was generated by quantifying the blots and normalizing the intensities of bands to the untreated lane.

JNK1/2 is a mitogen-activated by the upstream kinases such as TAK1 in response to stress^38,39^. While investigating the effect of ATE1 on TAK1, we observed that ATE1 depletion suppressed the phosphorylation of TAK1 Ligated ATE1 siRNA hearts as compared Ligated NS siRNA samples (Fig. 5A,B) Furthermore, our results demonstrated that the phosphorylation levels of JNK1/2 was increased after renal ligation surgery and ATE1 depletion in these rats significantly reduced the phosphorylation level of JNK1/2 but the levels of phosphorylated ERK1/2 remain unchanged (Fig. 5C–E). This, in turn, suggested that ATE1 knockdown act as a negative regulator of JNK1/2 signaling in ligated hearts. Hence, these data indicate that TAK1 activation was negatively regulated by ATE1 in response to renal artery ligation, which in turn controls the expression of hypertrophic genes. Collectively these results show that the antihypertrophic effect of ATE1 is associated with TAK1 mediated JNK1/2 signaling.

### ATE1 knockdown impairs the fibrosis and apoptosis

**Figure 6:**
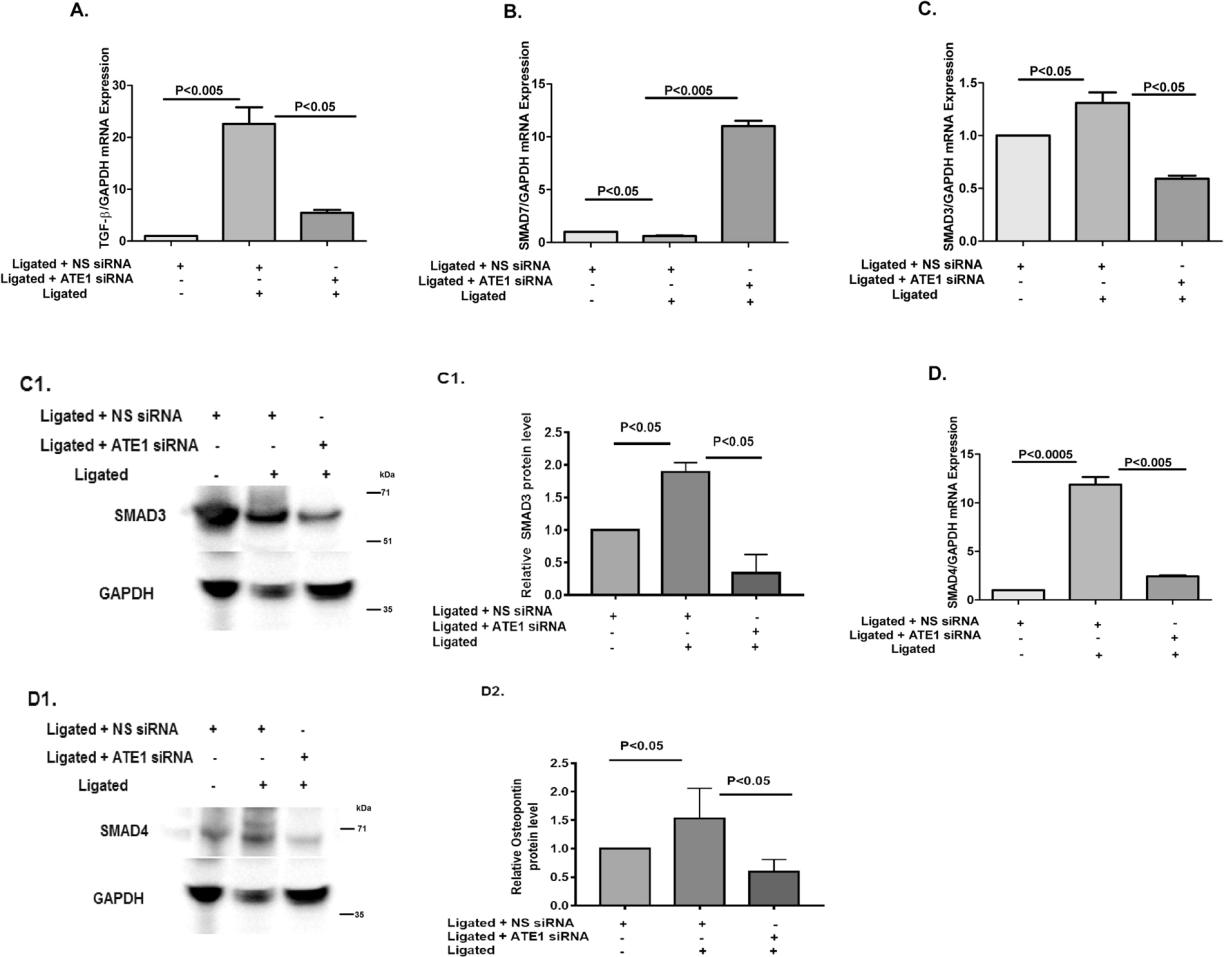
ATE1 knockdown impairs the TGF-β1 Smad signaling (**A**) TGF-β1 (**B**) Smad7 (**C**) Smad3 (**D**) Smad4 in (Ligated ATE1 siRNA) vs Ligated + NS siRNA (C1,D1) Western blot analysis of SMAD3 and SMAD4 protein levels in heart samples from (Ligated ATE1 siRNA) vs Ligated + NS siRNA (C2,D2) bar graph was generated by quantifying the blots and normalizing the intensities of bands to the untreated lane. Data derived from qRT-experiments performed in triplicate and normalized to GAPDH content. Data are represented as mean ± SE.

**Figure 7:**
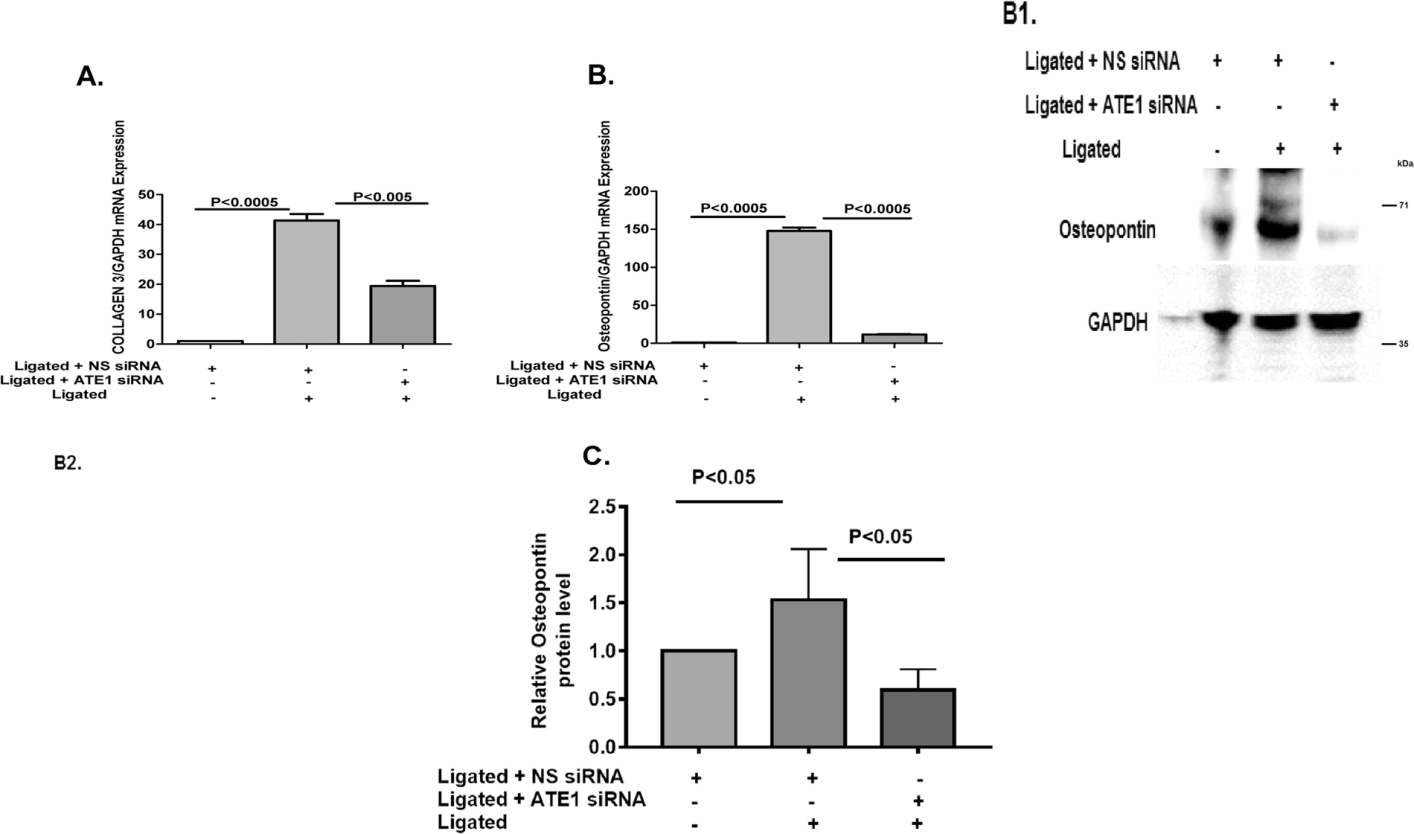
Quantitative real-time PCR analyses of fibrosis gene markers mRNA expression of (**A**) Collagen 3 (**B**) osteopontin (B1) Western blot analysis of osteopontin protein levels in heart samples from (Ligated ATE1 siRNA) vs Ligated + NS siRNA (B2) bar graph was generated by quantifying the blots and normalizing the intensities of bands to the untreated lane (**C**) CCN2 in the indicated groups. Data derived from independent experiments performed in triplicate and normalized to GAPDH content. Data are represented as mean ± SE.

Cardiac fibrosis is another important indication of pressure overload-induced pathological cardiac hypertrophy, which aggravates cardiac performance by the extension of the extracellular matrix ascribed to the accumulation of collagen^36^. Transforming growth factor-β (TGF-β) is a plays role crucial role in fibrosis^37^. To examine whether ATE1 modulate the classical fibrosis TGF-β1/Smad signaling pathway, TGF-β1 expression was checked and we noticed increased TGF-β1 mRNA expression following renal artery ligation and decreased in ATE1 suppressed state (Fig. 6A). The fibrotic effects of TGF-β1 are particularly caused by the Smad2/3-dependent pathway^40^. Further, an increase in Smad7 mRNA and protein level was observed in the case of ATE1 knockdown hypertrophied samples (Fig. 6B). Smad7 negatively regulate signal transduction by TGF-β1 by stably interacting with the activated TGF-β type I receptor, thereby preventing the binding to and activation of Smad2, 3 and thereafter 4^41,42^. The mRNA and protein expression level of Smad 3 and 4 was shown to be decreased in the case of ATE1 knockdown hypertrophied heart samples (Fig. 6C,C1,C2 & 6D,D1,D2). We also analyzed the synthesis of collagen by checking the expression of mRNA encoding collagen 3 (Fig. 7A), osteopontin (Fig. 7B) and CCN2 (Fig. 7C) which plays role in the proliferation of cardiac fibroblasts and the biosynthesis of ECM proteins. The expression of these markers was significantly lower in Ligated + ATE1siRNA as compared to Ligated + NS siRNA. Further, it indicates that ATE1 modulates the activation of cardiac fibrosis via the TGF-β1/Smad signaling pathway. We also checked protein expression levels of osteopontin, which was comparitively similar to mRNA levels (Fig. 7B1).

**Figure 8:**
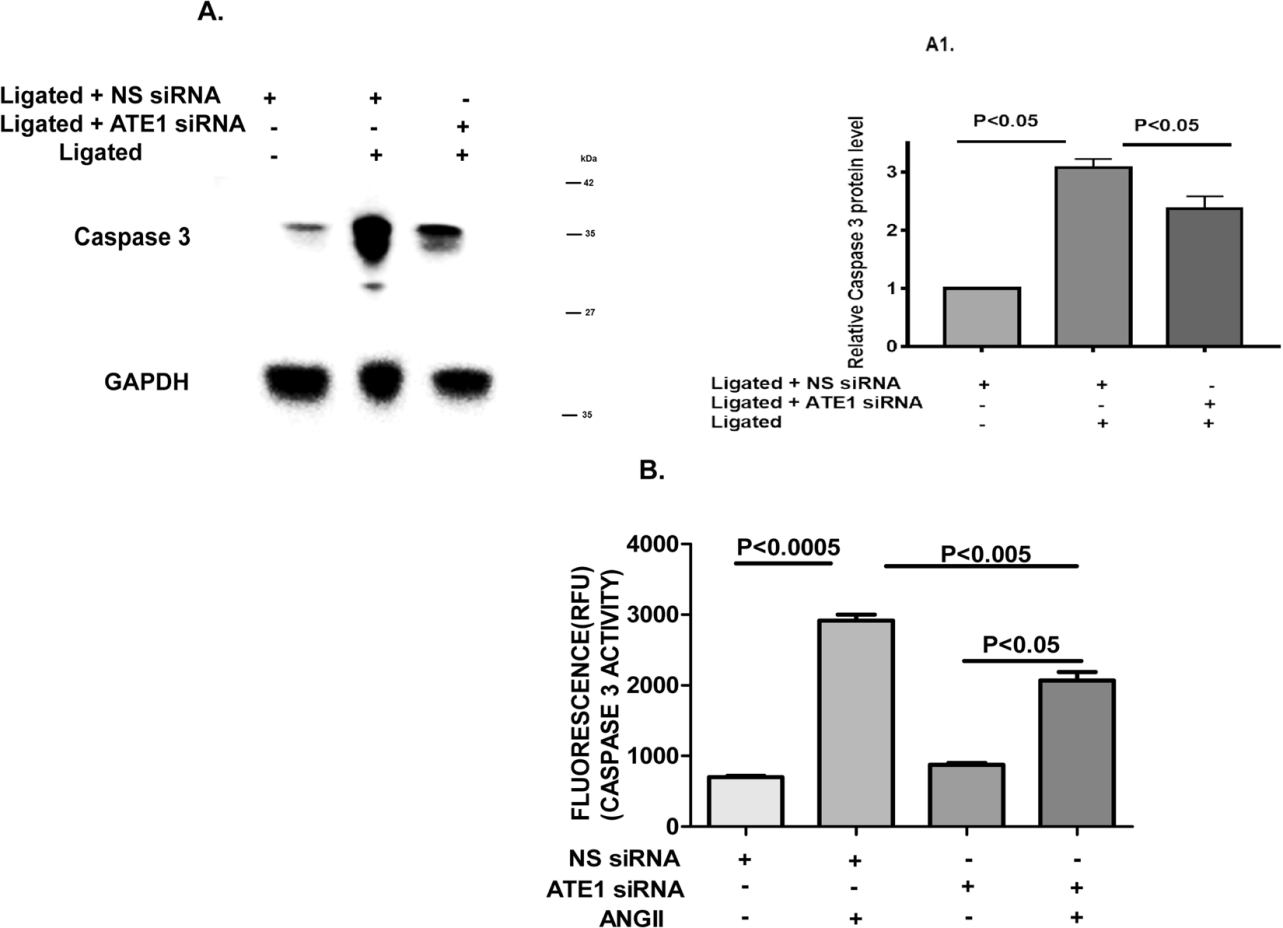
Knockdown of ATE1 promoted cardiac apoptosis (**A**,A1) Western and quantitative analysis of protein levels of active caspase 3 in Ligated ATE1 siRNA and Ligated NS siRNA. GAPDH was used as loading control. Bar graph was generated by quantifying the blots and normalizing the intensities of bands to the untreated lane. (**B**) Graph representing the fluorescence values of cells treated with and without Ang II in the ATE1 knockdown H9C2 samples.

Cardiac myocyte apoptosis has an utmost crucial role in the progression of cardiac hypertrophy to heart failure. To determine whether ATE1 knockdown hypertrophied rat samples play importance role in apoptotic signals, we analyzed the expression of caspase 3 (Fig. 8). As expected, these displayed a reduction of caspase 3 expression in response to Ligated + ATE1siRNA rats. We also confirmed it with fluorescence-based Caspase-3/7 Assay as described in the method section. Here, the amount of fluorescent product generated is proportional to the amount of caspase-3/7 cleavage activity present in the sample. We found the relative fluorescent unit (RFU) to be lesser in the case of cells undergone Ang II treatment with ATE1 siRNA (Figure 8B). Hence the knockdown of ATE1 in case of cardiac stress helps the cells to reduce the activation of programmed cell death cascade and thus lowering down pressure induced cardiac apoptosis.

## Discussion

ATE1 is an evolutionary conserved enzyme, which is present in different species^43,44^. Due to alternative splicing, mammalian species contain more than one form of ATE1^45^. This gene has a significant role in multiple biological events and many aspects are yet to be discovered. Our work addresses the novel role of ATE1 in presence of cardiac hypertrophic stress in adult rats. We show that ATE1 is upregulated in response to cardiac stress in adult rats and its deficiency in such heart cells results in the prevention of expression of genes regulating cardiac hypertrophy cum fibrosis and apoptosis. Our data clarify the involvement of ATE1 in case of stress, potentially providing a novel insight for the *in vivo* functions of ATE1 in case of cardiac diseases. In response to cardiac stress, the heart undergoes compensatory hypertrophic enlargement characterized by an increase in the cellular mass of individual adult cardiomyocytes^46^. However persistent workload leads to cardiac fibrosis and loss of myocytes by apoptosis ultimately leading to heart failure^47,48^. ATE1 is known to have a role in normal development and functioning of cardiovascular system^18,21^ and interestingly our study identified that expression of ATE1 was upregulated in case of cardiac stress both *invitro* and *invivo*. Biochemical and genetic evidence have earlier shown that reduced expression of ATE1 is correlated with cardiac disorders in embryonic stages^18,19^. To gain an insight into the regulatory role of ATE1, we knockdown ATE1 using siRNA-based transfection in H9C2 cells induced by Ang II. Results indicated that decreased expression of endogenous ATE1 attenuated the hypertrophic effect of Ang II. To further potentiate it in *invivo*, we generated renal artery ligated rats with cardiac specific knockdown of ATE1. Consistent with the *invitro* data, ATE1 deficiency protected cardiac hypertrophy. Further ATE1 knockdown has improved the ventricular dilation and contractile dysfunction in Ligated + ATE1 siRNA rats. Also, we have seen from our results that correlation between mRNA and protein level is poor. As per previous studies mRNA and protein correlation depends on various biological and technical factors viz. protein half-life, Post-translational modifications, and experimental errors etc^49^.

An earlier study shows that ATE1 knockout mice led to defective cardiovascular development and angiogenesis partially because of deregulated Gαq-PLC/PKC-MEK1-ERK signaling^20^. Interestingly, our results indicated another potential involvement of ATE1 in cardiac diseases. According to our results, ATE1 knockdown exerted antihypertrophic effects by suppressing the activation of TAK1 signaling. Activation of TAK1 plays a role in inducing cardiac hypertrophy in the presence of stress suggesting it to be crucial for maintaining cardiac homeostasis^50,51^. Our data indicated that ATE1 depletion reduced TAK1 activation in response to hypertrophic stress. TAK1 is an upstream kinase for ERK1/2 and JNK1/2 signaling^52,53^ (Figure S12). Further, the phosphorylation level of JNK1/2 was found to be decreased in ATE1 knockdown state but ERK1/2 levels remain constant, stating this ATE1 deficiency to be affecting the downstream factors of TAK1 which further controls the expression of hypertrophy genes. Interestingly, we observed that ATE1 knockdown further protected the heart cells from the deleterious effects of cardiac fibrosis and apoptosis. Cardiac fibrosis is an important mediator of heart function and is characterized by the amassing of extracellular matrix and collagen^54^. The present study showed decreased fibrosis in ATE1 knockdown hypertrophied rats via TGFβ1-Smad signaling (Figure S12). mRNA expression of TGF-β, a key mediator of fibrosis was found to be decreased in case of ATE1 knockdown hypertrophied rats^55^. Decreased Smad3/4 mRNA level and increased Smad7 mRNA levels when ATE1 is knocked down in renal artery ligated rat samples indicates that ATE1 is playing a crucial role in regulating this pathway. Furthermore, the reduced protein expression of fibrotic markers like Collagen 3, and Osteopontin in response to hypertrophic stimuli in ATE1 knockdown state justifies the role of ATE1 in cardiac fibrosis as well. Cardiac myocyte apoptosis plays a crucial role in the transition of cardiac hypertrophy to heart failure^56^. We examined the role of ATE1 in regulating cardiomyocyte survival. The present study demonstrated the cardiomyocyte protective function of ATE1. We found that knockdown of ATE1 attenuates myocardial apoptosis and is associated with abrogated expression of caspase3 (Figure S12). Further investigation is required to elucidate the mechanism of apoptosis and its relation to ATE1 knockdown. As per the previous studies, the intracellular levels of ATE1 are maintained in the cells and a sudden decrease or any fluctuation of its expression causes several diseases. Our findings show that ATE1 is upregulated as part of the cardiac stress response and its knockdown further leads to protect the cardiac cells from further damage. It is worth pointing out that many regulatory proteins/factors must be involved to increase this ATE1 expression, information about those is not covered here.Further studies would be required to study such individual factors contributing to increase ATE1 level in stress state. Although our data suggest that the knockdown of ATE1 plays a role in cardio protection in adult Rat model, we cannot dismiss possible involvement of other potential regulatory proteins here.

In summary, the present work demonstrates that ATE1 may be a critical endogenous regulator of cardiac remodeling and dysfunction. Here, we demonstrated that the knockdown of ATE1 in the presence of cardiac stress protects against cardiac hypertrophy. The mechanism underlying the protective effects of ATE1 knockdown appears to involve the inhibition of the TAK1-JNK1/2 signaling pathway. Our results also shed a light on the role of ATE1 in regulating the expression of fibrotic gene markers by regulating TGF-β-smad signaling as well as cardiac apoptosis. These findings suggest that ATE1 act as a regulator of hypertrophic response and may be used as a therapeutic target for cardiac hypertrophy and fibrosis^57–62^.

## Supplementary information

Supplementary Information

## References

[1] Savarese G, Lund LH (2017). Global public health burden of heart failure. Card. Fail. Rev..

[2] Ambrosy AP (2014). The global health and economic burden of hospitalizations for heart failure: lessons learned from hospitalized heart failure registries. J. Am. Coll. Cardiol..

[3] Roger VL (2013). Epidemiology of heart failure. Circ. Res..

[4] Tham YK, Bernardo BC, Ooi JYY, Weeks KL, McMullen JR (2015). Pathophysiology of cardiac hypertrophy and heart failure: signaling pathways and novel therapeutic targets. Arch. Toxicol..

[5] Heinzel FR, Hohendanner F, Jin G, Sedej S, Edelmann F (2015). Myocardial hypertrophy and its role in heart failure with preserved ejection fraction. J. Appl. Physiol..

[6] Rame JE, Dries DL (2007). Heart failure and cardiac hypertrophy. Curr. Treat. Options Cardiovasc. Med..

[7] Stansfield, W. E. *et al*. The Pathophysiology of Cardiac Hypertrophy and Heart Failure. *In Cellular and Molecular Pathobiology of Cardiovascular Disease*, 51–78 (2014).

[8] Tardiff JC (2006). Cardiac hypertrophy: Stressing out the heart. J. Clin. Invest..

[9] Nakamura M, Sadoshima J (2018). Mechanisms of physiological and pathological cardiac hypertrophy. Nat. Rev. Cardiol.

[10] Shimizu I, Minamino T (2016). Physiological and pathological cardiac hypertrophy. Journal of Molecular and Cellular Cardiology.

[11] Creemers EE, Pinto YM (2011). Molecular mechanisms that control interstitial fibrosis in the pressure-overloaded heart. Cardiovasc. Res..

[12] Fan D, Takawale A, Lee J, Kassiri Z (2012). Cardiac fibroblasts, fibrosis and extracellular matrix remodeling in heart disease. Fibrogen. Tissue Repair.

[13] Kong P, Christia P, Frangogiannis NG (2014). The pathogenesis of cardiac fibrosis. Cell. Mol. Life Sci..

[14] Ho CY (2010). Myocardial fibrosis as an early manifestation of hypertrophic cardiomyopathy. N. Engl. J. Med..

[15] Birnbaum MD (2019). Reduced Arginyltransferase 1 is a driver and a potential prognostic indicator of prostate cancer metastasis. HHS Public Access..

[16] Wang, J. *et al*. Protein arginylation targets alpha synuclein, facilitates normal brain health, and prevents neurodegeneration. *Sci. Rep*. 1–14, 10.1038/s41598-017-11713-z (2017). 10.1038/s41598-017-11713-z PMC5595787 28900170

[17] Wang J (2017). Arginyltransferase ATE1 is targeted to the neuronal growth cones and regulates neurite outgrowth during brain development. HHS Public Access..

[18] Kwon YT (2002). An essential role of N-terminal arginylation in cardiovascular development. Science.

[19] Rai R (2008). Arginyltransferase regulates alpha cardiac actin function, myofibril formation and contractility during heart development. Development.

[20] Lee MJ (2012). Characterization of arginylation branch of N-end rule pathway in G-protein-mediated proliferation and signaling of cardiomyocytes. J. Biol. Chem..

[21] Kurosaka S (2012). Arginylation regulates myofibrils to maintain heart function and prevent dilated cardiomyopathy. J. Mol. Cell. Cardiol..

[22] Ribeiro PAB (2013). Contractility of myofibrils from the heart and diaphragm muscles measured with atomic force cantilevers: Effects of heart-specific deletion of arginyl-tRNA-protein transferase. Int. J. Cardiol..

[23] Maier T, Güell M, Serrano L (2009). Correlation of mRNA and protein in complex biological samples. FEBS Lett..

[24] Ditzel M (2003). Degradation of DIAP1 by the N-end rule pathway is essential for regulating apoptosis. Nat. Cell Biol..

[25] Deka K, Saha S (2017). Arginylation: a new regulator of mRNA stability and heat stress response. Cell Death Dis..

[26] Datta Ritwik (2017). Myocyte-derived Hsp90 modulates collagen upregulation via biphasic activation of STAT-3 in fibroblasts during cardiac hypertrophy. Mole. Cell. Biol..

[27] Chatterjee A (2006). Analysis of p53 and NF‐κB signaling in modulating the cardiomyocyte fate during hypertrophy. J. cell Physiol..

[28] Sen S (1974). Cardiac hypertrophy in spontaneously hypertensive rats. Circ. Res..

[29] Rana S (2000). A spatio-temporal cardiomyocyte targeted vector system for efficient delivery of therapeutic payloads to regress cardiac hypertrophy abating bystander effect. J. Control. Release.

[30] Shim JH (2005). TAK1, but not TAB1 or TAB2, plays an essential role in multiple signaling pathways *in vivo*. Genes Dev..

[31] Johnson, G. L. & Lapadat, R. Mitogen-activated protein kinase pathways mediated by ERK, JNK, and p38 protein kinases. *Science* **298**, 1911-1912 (2002). 10.1126/science.1072682 12471242

[32] Pearson G (2001). Mitogen-activated protein (MAP) kinase pathways: Regulation and physiological functions. Endocrinol Rev..

[33] Qi M, Elion Ea (2005). MAP kinase pathways. J. Cell Sci..

[34] Muslin AJ (2008). MAPK signalling in cardiovascular health and disease: molecular mechanisms and therapeutic targets. Clin. Sci..

[35] Wang Y (2007). Mitogen-activated protein kinases in heart development and diseases. Circulation.

[36] Sugden PH, Clerk A (1998). ‘Stress-responsive’ mitogen-activated protein kinases (c-Jun N-terminal kinases and p38 mitogen-activated protein kinases) in the myocardium. Circ. Res..

[37] Molkentin JD, Dorn GW (2001). Cytoplasmic Signaling Pathways That Regulate Cardiac Hypertrophy. Annu. Rev. Physiol..

[38] Pattison MJ (2016). TLR and TNF-R1 activation of the MKK3/MKK6-p38 axis in macrophages is mediated by TPL-2 kinase. Biochem. J..

[39] Sabio G, Davis RJ (2014). TNF and MAP kinase signalling pathways. Semin. Immunol..

[40] Biernacka A, Dobaczewski M, Frangogiannis NG (2011). TGF-β signaling in fibrosis. Growth Factors.

[41] Park SH (2005). Fine tuning and cross-talking of TGF-beta signal by inhibitory Smads. J. Biochem Mol. Bio..

[42] Lönn P, Morén A, Raja E, Dahl M, Moustakas A (2009). Regulating the stability of TGFβ receptors and Smads. Cell Res..

[43] Balzi E, Choder M, Chen WN, Varshavsky A, Goffeau A (1990). Cloning and functional analysis of the arginyl-tRNA-protein transferase gene ATE1 of Saccharomyces cerevisiae. J. Biol. Chem..

[44] Graciet E (2006). Aminoacyl-transferases and the N-end rule pathway of prokaryotic/eukaryotic specificity in a human pathogen. Proc. Natl. Acad. Sci..

[45] Kwon YT, Kashina AS, Varshavsky A (1999). Alternative splicing results in differential expression, activity, and localization of the two forms of arginyl-tRNA-protein transferase, a component of the N-end rule pathway. Mol. Cell. Biol..

[46] Waring CD (2012). The adult heart responds to increased workload with physiologic hypertrophy, cardiac stem cell activation, and new myocyte formation. Eur. Heart J..

[47] Chiong M (2011). Cardiomyocyte death: Mechanisms and translational implications. Cell Death Dis..

[48] Piek A, de Boer RA, Silljé HHW (2016). The fibrosis-cell death axis in heart failure. Heart Fail. Rev..

[49] Maier T, Güell M, Serrano L (2009). Correlation of mRNA and protein in complex biological samples. FEBS Lett..

[50] Li L (2014). Transforming growth factor βactivated kinase 1 signaling pathway critically regulates myocardial survival and remodeling. Circulation.

[51] Zhang D (2000). TAK1 is activated in the myocardium after pressure overload and is sufficient to provoke heart failure in transgenic mice. Nat. Med..

[52] Zhang J, Macartney T, Peggie M, Cohen P (2017). Interleukin-1 and TRAF6-dependent activation of TAK1 in the absence of TAB2 and TAB3. Biochem. J..

[53] Sato S (2005). Essential function for the kinase TAK1 in innate and adaptive immune responses. Nat. Immunol..

[54] Cai J (2009). Targeted expression of receptor-associated late transducer inhibits maladaptive hypertrophy via blocking epidermal growth factor receptor signaling. Hypertension.

[55] Mauviel A (2005). Transforming growth factor-beta: a key mediator of fibrosis. Methods Mol.Med..

[56] Wencker D (2003). A mechanistic role for cardiac myocyte apoptosis in heart failure. J. Clin. Invest..

[57] Ninomiya-Tsuji J (1999). The kinase TAK1 can activate the NIK-IκB as well as the MAP kinase cascade in the IL-1 signalling pathway. Nature.

[58] Wang C (2001). TAK1 is a ubiquitin-dependent kinase of MKK and IKK. Nature.

[59] Shirakabe K (1997). TAK1 mediates the ceramide signaling to stress-activated protein kinase/c-Jun N-terminal kinase. J. Biol. Chem..

[60] Shim JH (2009). TAK1 is an essential regulator of BMP signalling in cartilage. EMBO J..

[61] Arthur JSC, Ley SC (2013). Mitogen-activated protein kinases in innate immunity. Nat. Rev. Immunol..

[62] Zhang J, Clark K, Lawrence T, Peggie MW, Cohen P (2014). An unexpected twist to the activation of IKKβ: TAK1 primes IKKβ for activation by autophosphorylation. Biochem. J..

