# Author Correction: Arginyltransferase knockdown attenuates cardiac hypertrophy and fibrosis through TAK1-JNK1/2 pathway

**DOI:** 10.1038/s41598-020-67556-8

**Published:** 2020-06-25

**Authors:** Kanika Singh, Ankit Gupta, Ashish Sarkar, Ishita Gupta, Santanu Rana, Sagartirtha Sarkar, Sameena Khan

**Affiliations:** 1 Drug Discovery Research Centre, Translational Health Science and Technology Institute, Faridabad, Haryana India; 2 Department of Zoology, University of Calcutta, Kolkata, India; 3 Structural Immunology Group, International Centre for Genetic Engineering and Biotechnology, New Delhi, Delhi India

Correction to:* Scientific Reports* https://doi.org/10.1038/s41598-019-57379-7, published online 17 January 2020

This Article contains errors.

In Figure 5A, the orientation of GADPH blot for P-TAK1 is incorrect. In addition, width of Figure 5B has been adjusted. The correct Figure 5 appears below, as Figure 1.

**Figure 1:**
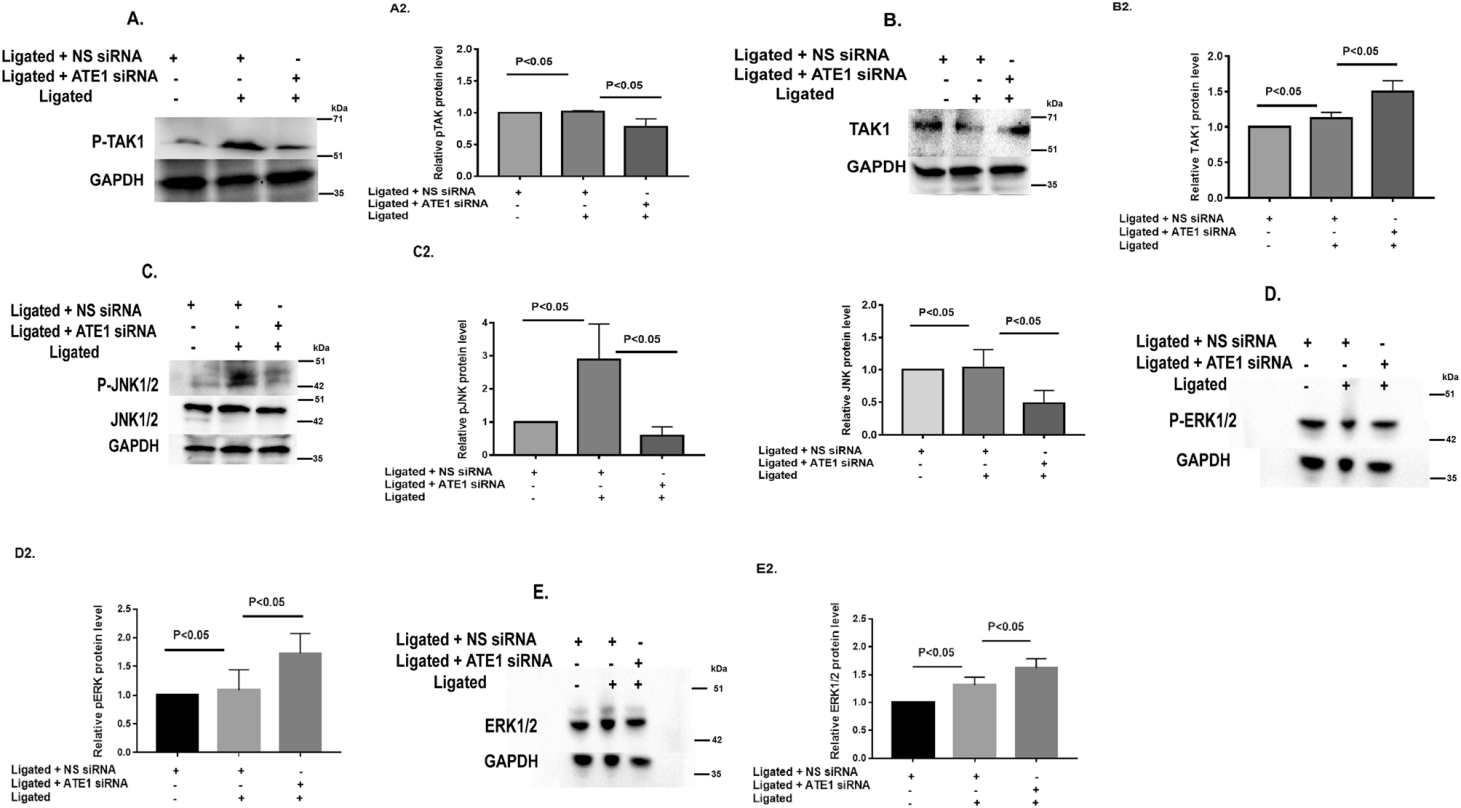
.

In Figure 6D1, the GADPH blot for SMAD4 was inadvertently duplicated from the SMAD3 western. In addition, the graph for Figure 6D2 is incorrect. The correct Figure 6 appears below, as Figure 2.

**Figure 2:**
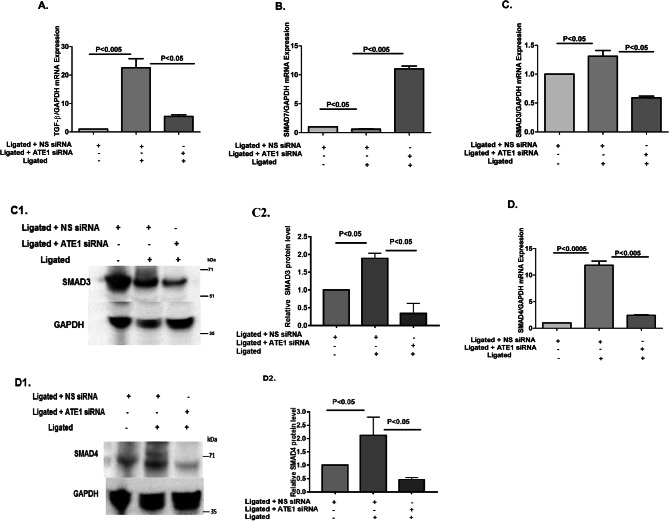
.

In Supplementary Figure S9, the GADPH blot for SMAD4 was inadvertently duplicated from the SMAD3 western. The correct Figure S9 appears below, as Figure 3.

**Figure 3:**
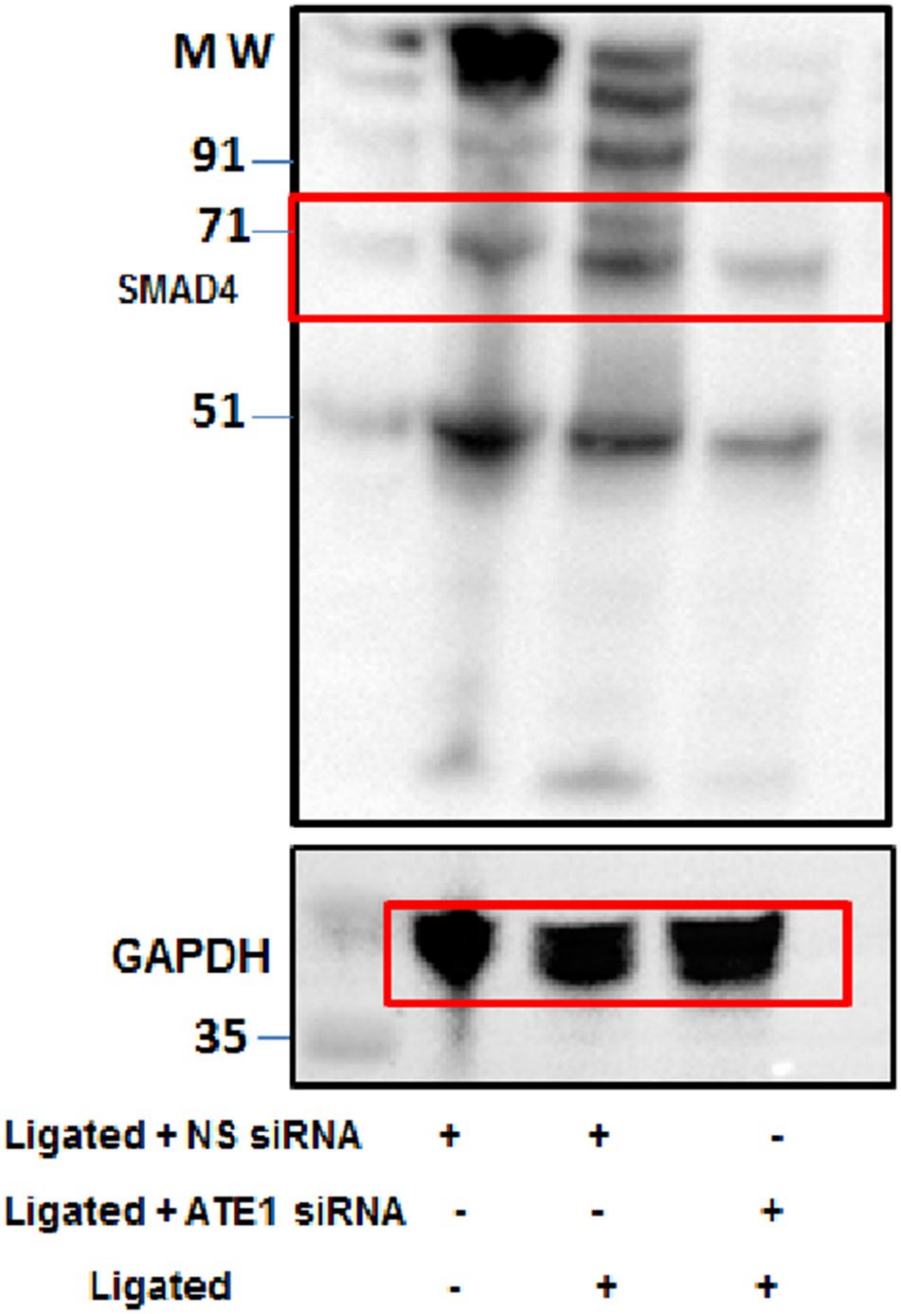
.

In Figure 7, Figure 7B2 is presented as Figure 7C, and no graph is shown for Figure 7B2. The correct Figure 7 appears below, as Figure 4.

Finally in the legend of Figure 8, the text,

**Figure 4:**
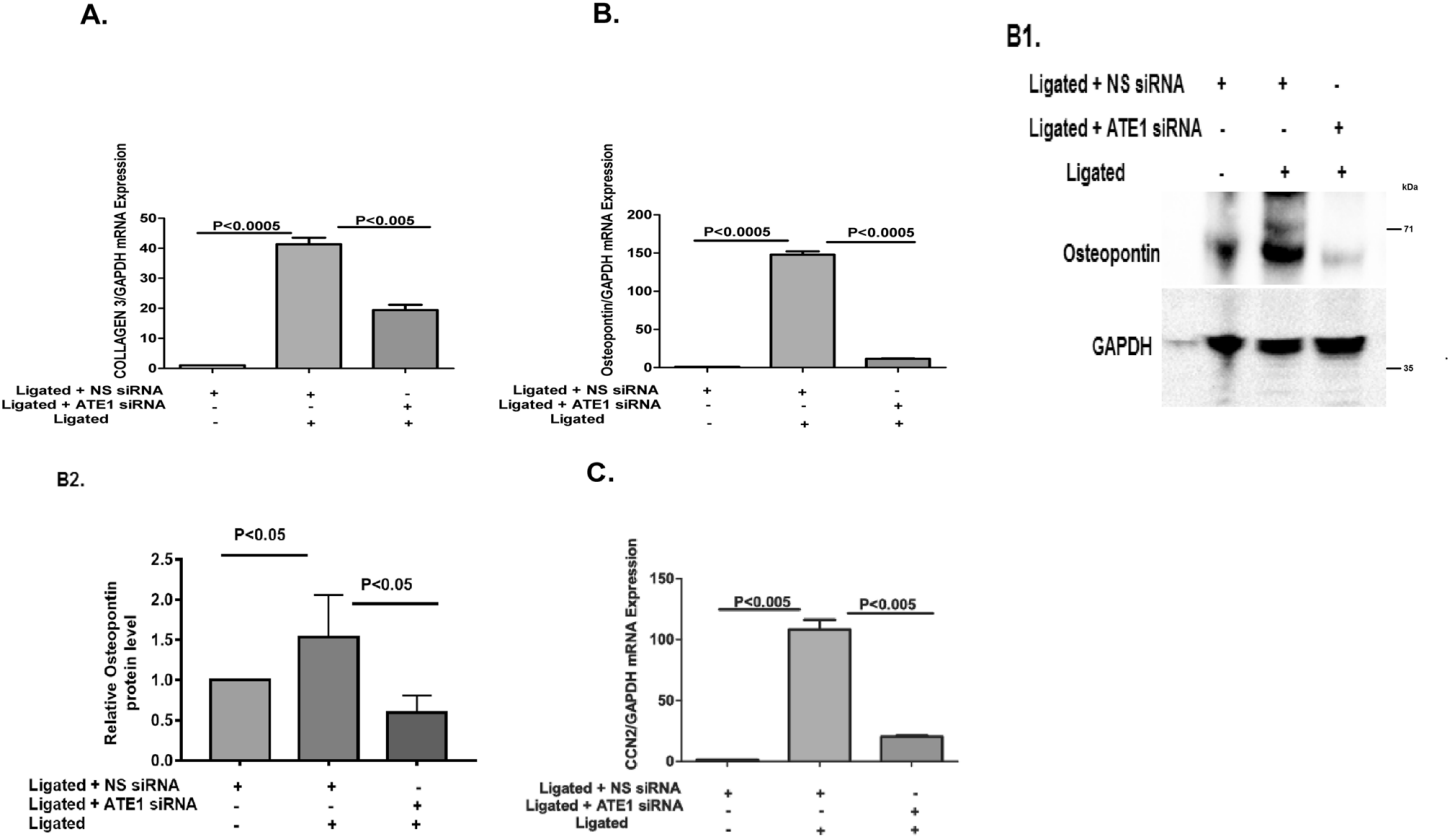
.

“Knockdown of ATE1 promoted cardiac apoptosis”.

should read:

“Knockdown of ATE1 mitigates cardiac apoptosis”.

