# Supplementary material for: Arginyltransferase knockdown attenuates cardiac hypertrophy and fibrosis through TAK1-JNK1/2 pathway
Source: Sci Rep. 2020 Jan 17;10:598. doi: 10.1038/s41598-019-57379-7 (PMC6969214; doi:10.1038/s41598-019-57379-7)
Supplement: Supplementary file 1 — Supplementary Information [file 41598_2019_57379_MOESM1_ESM.pdf]

Arginyltransferase knockdown attenuates cardiac hypertrophy and fibrosis through TAK1-JNK1/2 pathway

Kanika Singh#[1], Ankit Gupta#[1], Ashish Sarkar[1], Ishita Gupta[1,3], Santanu Rana[2], Sagartirtha Sarkar[2], Sameena Khan*[1]

[1] Drug Discovery Research Centre, Translational Health Science and Technology Institute, Faridabad, Haryana, India.

[2] Department of Zoology, University of Calcutta, Kolkata, India.

[3] Structural Immunology Group, International Centre for Genetic Engineering and Biotechnology, New Delhi, Delhi, India.

#contributed equally

[*]Correspondence:

Sameena Khan

sameena@thsti.res.in

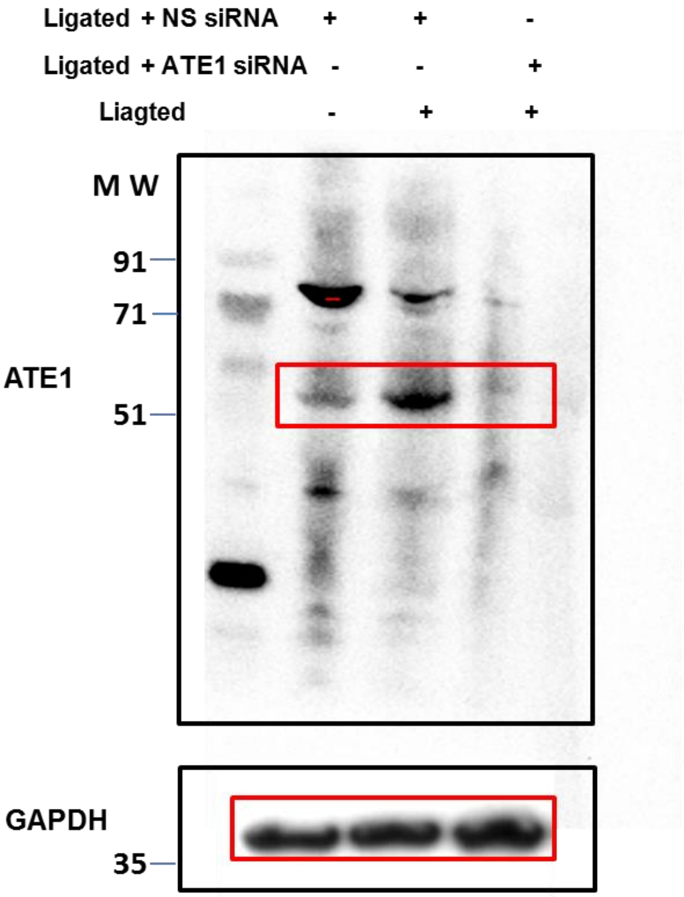

Figure S1

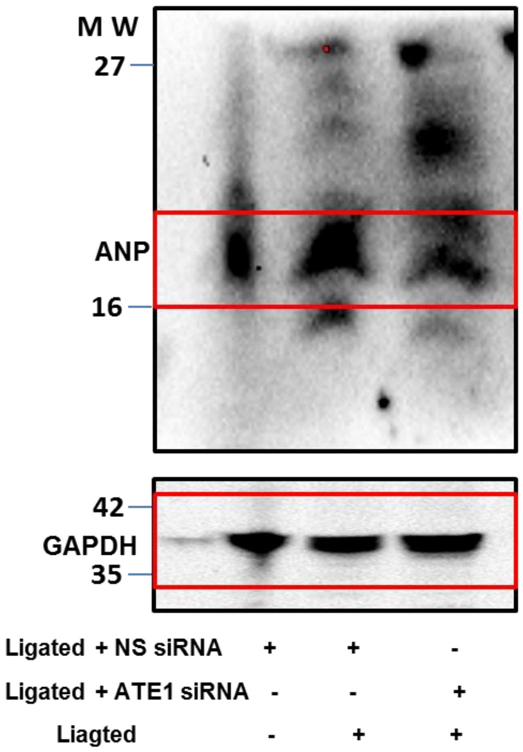

Figure S2

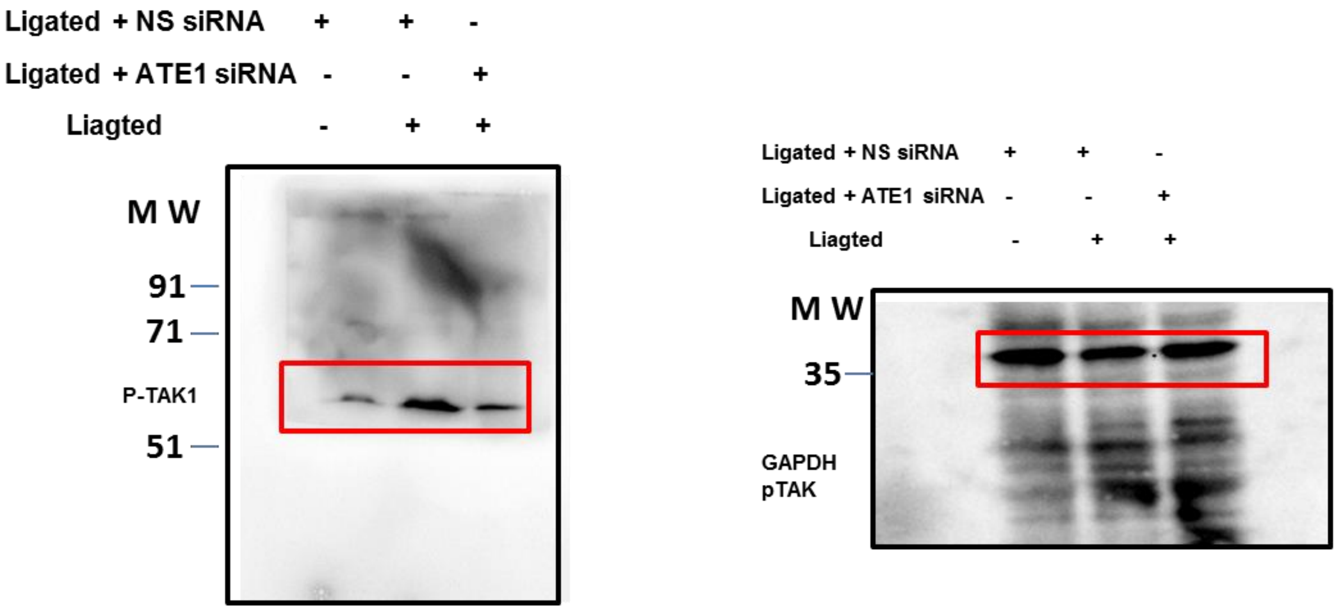

Figure S3

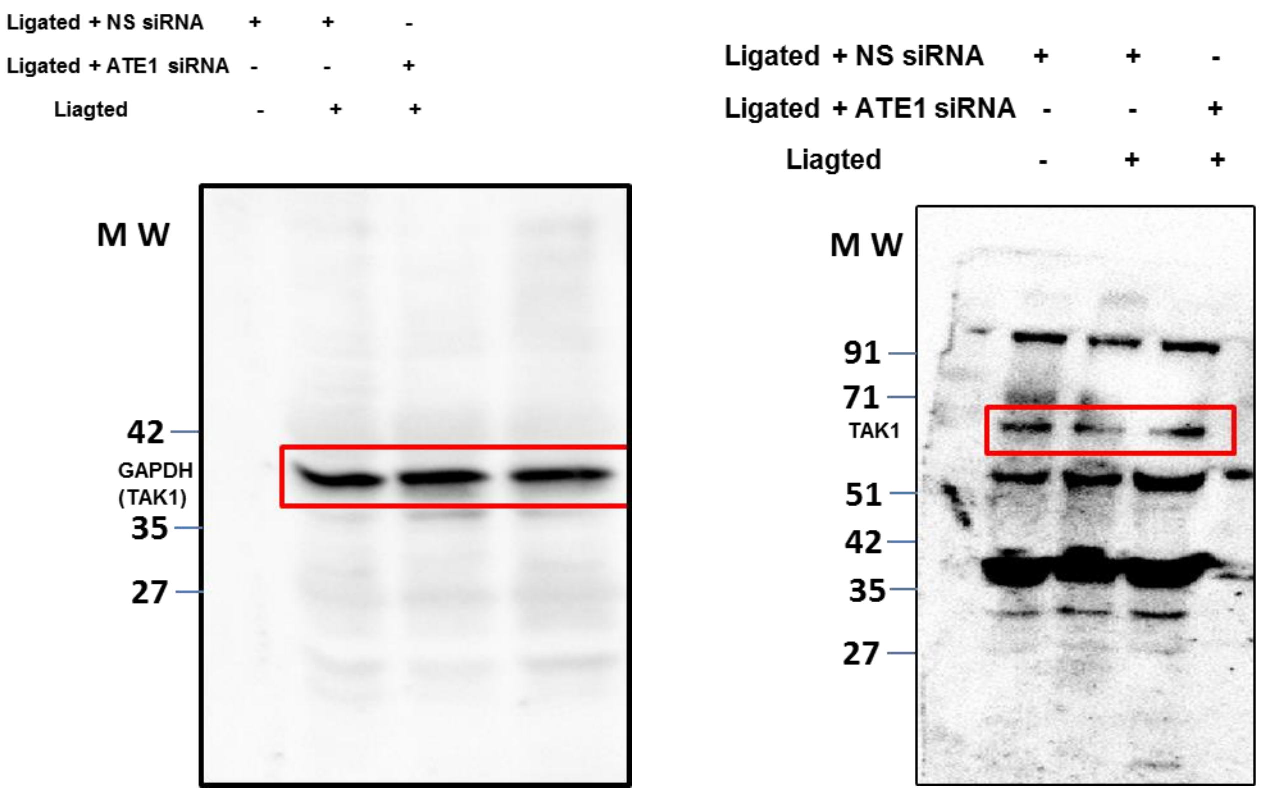

Figure S4

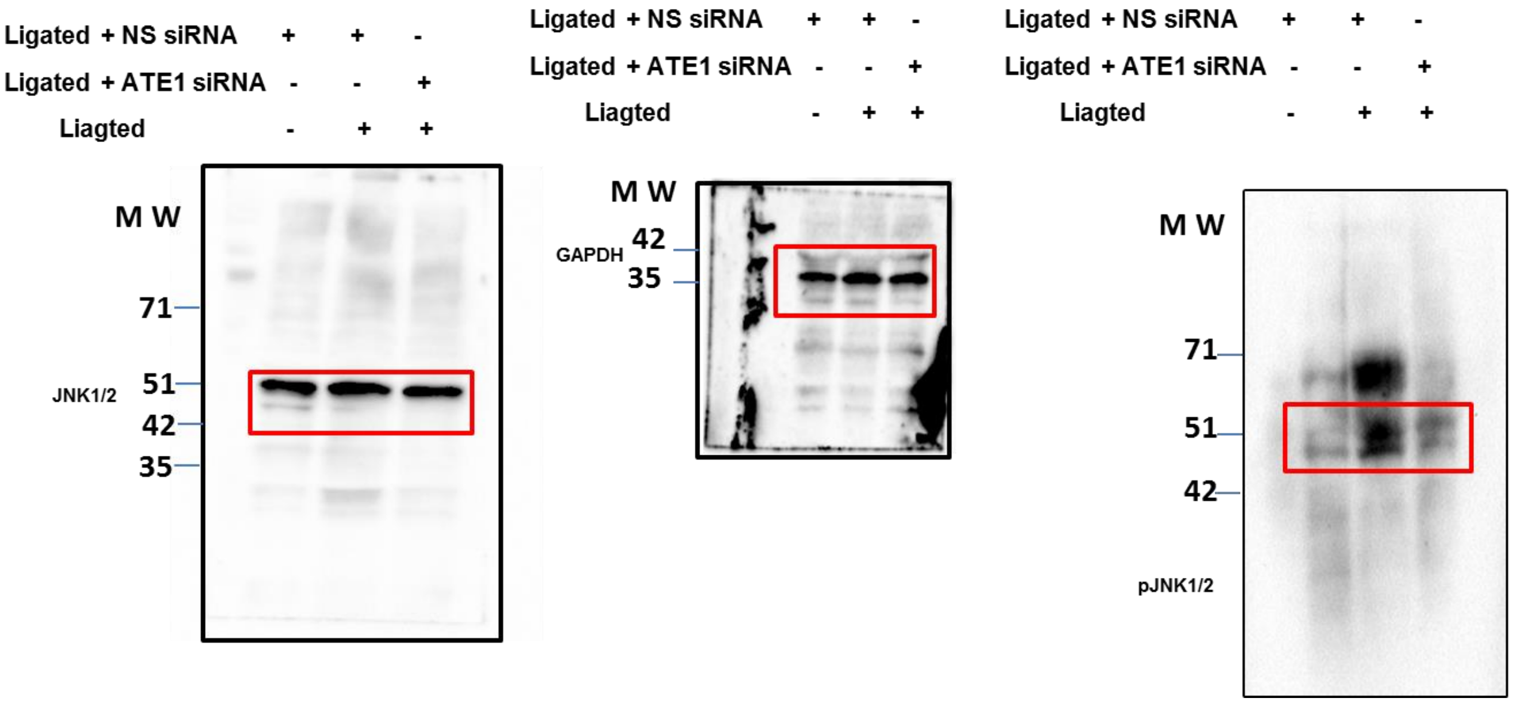

Figure S5

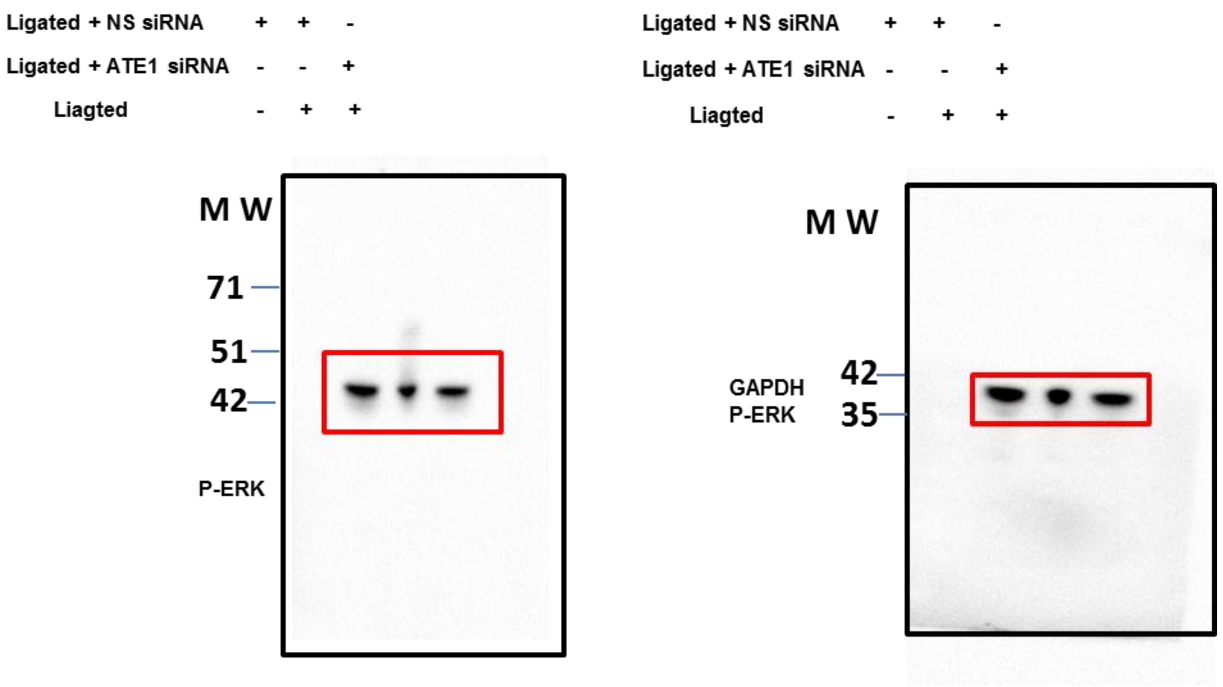

Figure S6

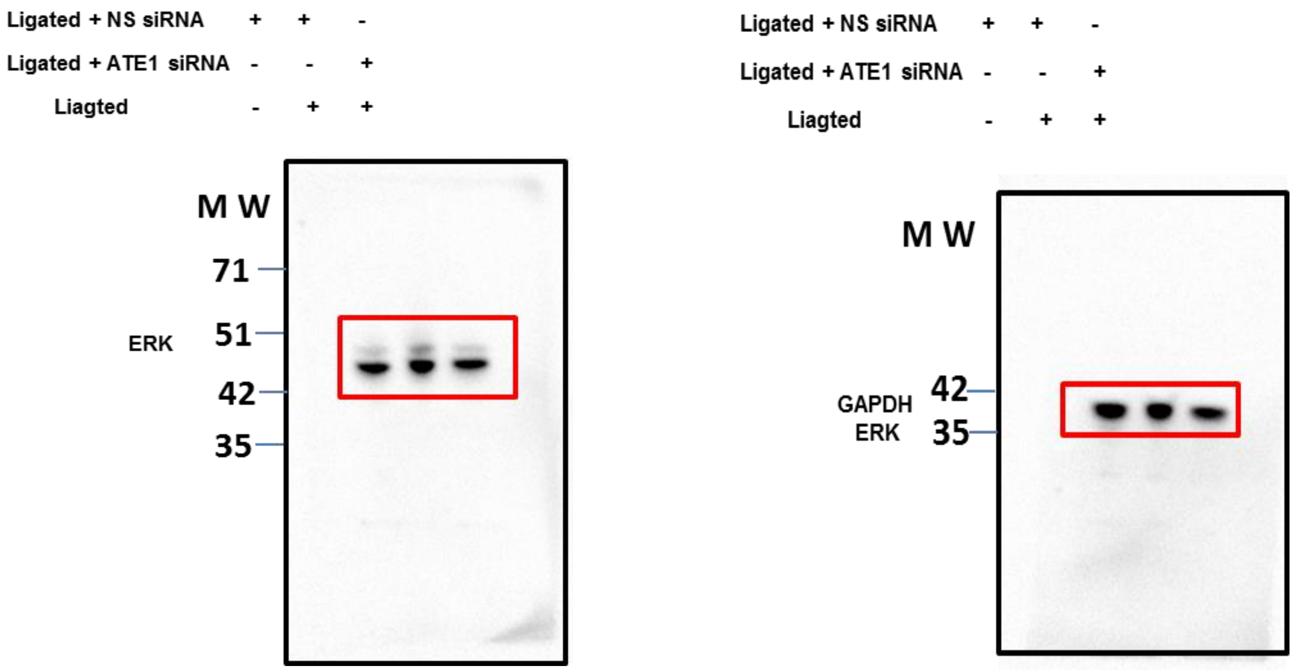

Figure S7.

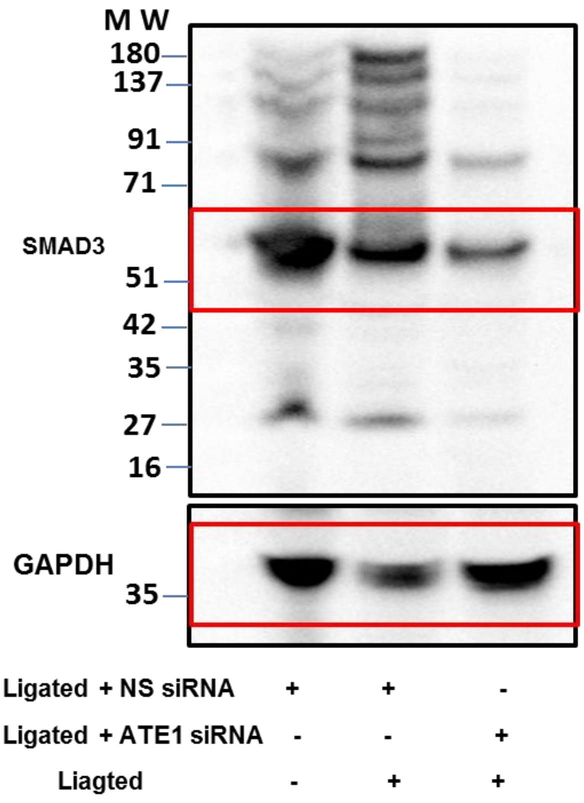

Figure S8

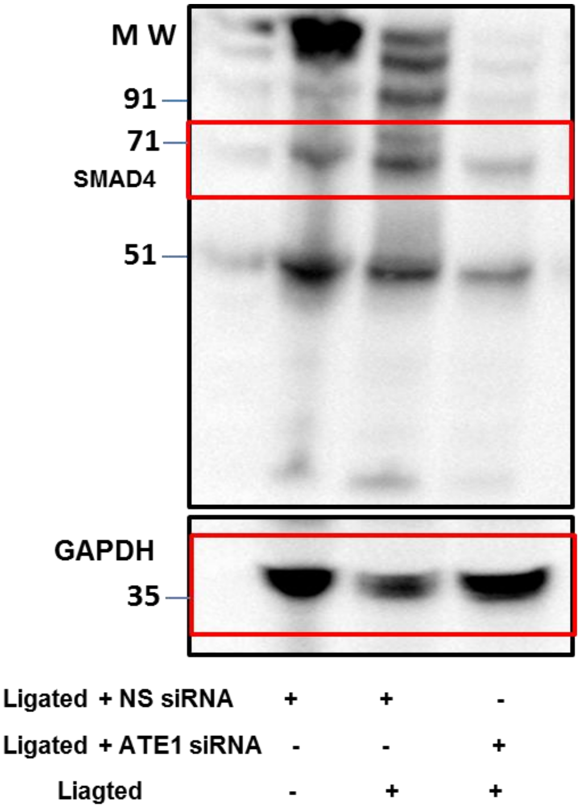

Figure S9

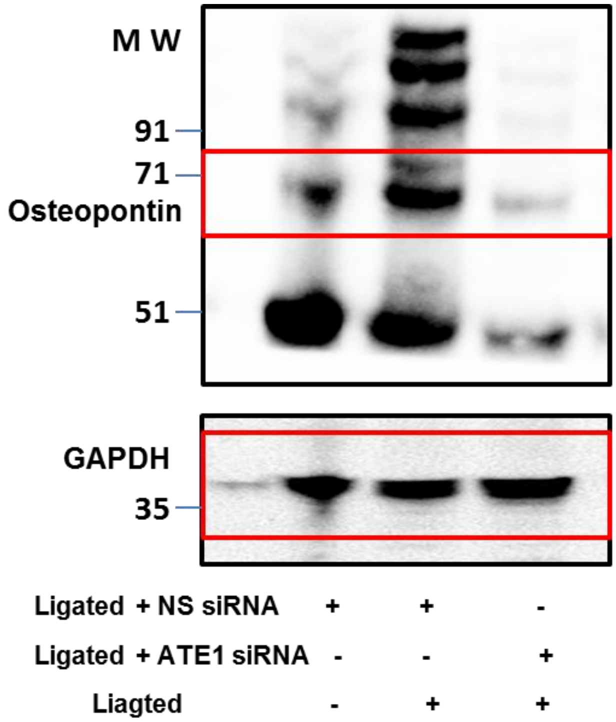

Figure S10

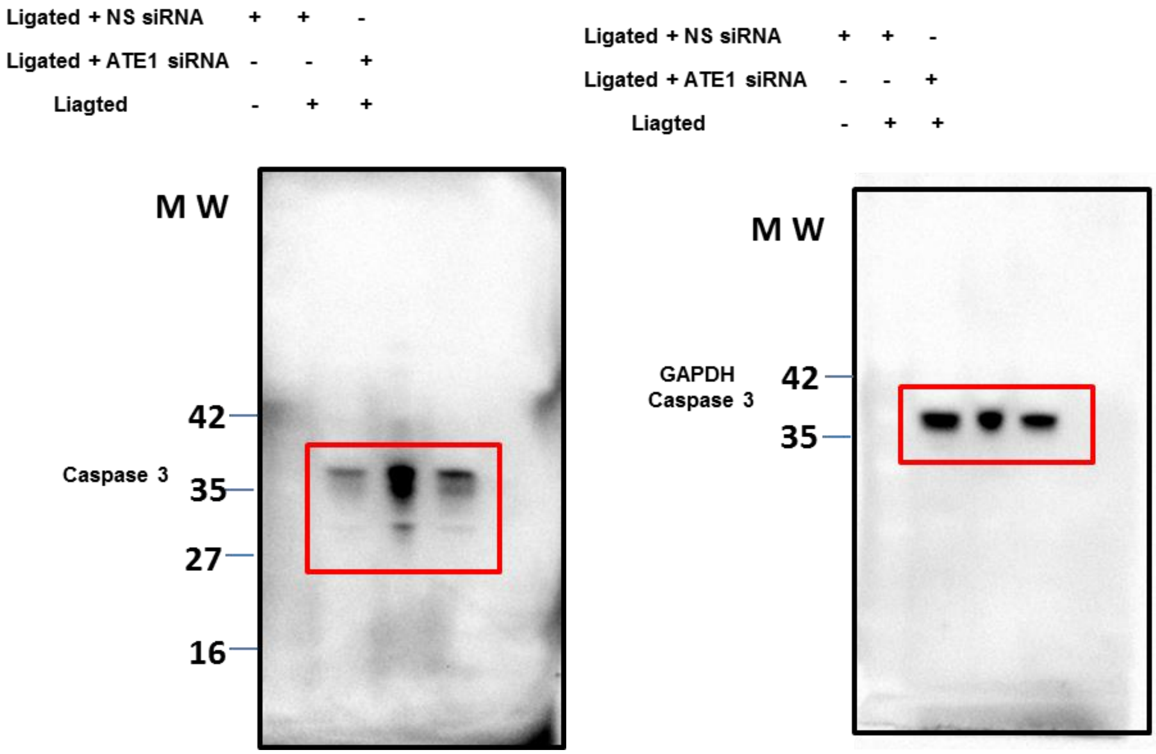

Figure S11

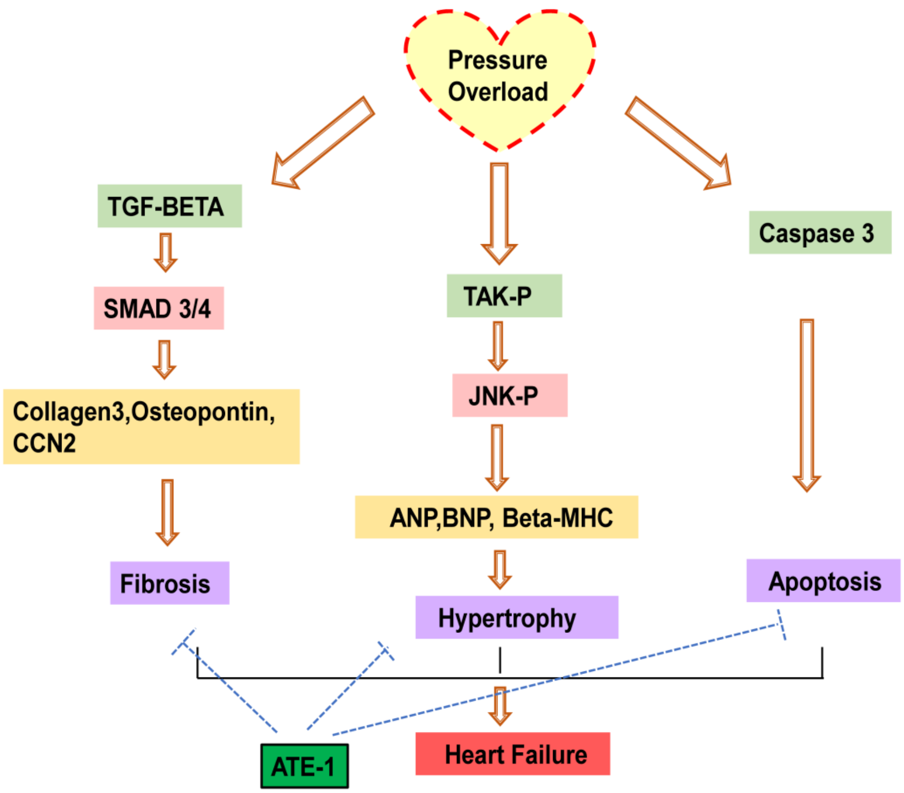

Figure S12 Cartoon representation of the summary of ATE1 role in regulating cardiac hypertrophy. In the present study, we demonstrated that the cardiac function can be improved by the reduced expression levels of ATE1 and that can be achieved by blocking three proposed signaling pathways. First, activated TAK1 results in activation of JNK1/2 signaling pathway, enhances hypertrophic markers expression, and subsequently leads to cardiac hypertrophy Second, TGFβ-Smad signaling activated due to pressure overload, increase expression of fibrotic markers and result in collagen synthesis. Third, hypertrophic stimuli also activate apoptotic signaling, ultimately leading to apoptosis. ATE1 knockdown blocks the TAK dependent cardiac hypertrophy pathway and TGFβ-Smad signaling dependent cardiac fibrosis. This knockdown also blocks caspase 3 activation

Table S1. Primers used for real-time PCR analysis

Gene	Forward Primer	Reverse Primer
ATE1	CTTACCAGGTCTTATAAACGTTAC	TTGTATCTCATCTTGGGACAGGA
ANP	CTCCGATAGATCTGCCCTCTTGAA	GGTACCGGAAGCTGTTGCAGCCTA
BNP	TCAGAGGAAATGGCTCAGAGACAG	GCA GGC AGA GTC AGA AGC CGG A
β-MHC	AAG GCC AAG ATC GTG TCT CGA GA	ACA CAG AAG AGG CCT GAG TAG GTG
TGF-β	GCTTCAGTGCTCACTGCTCTTGT	GTACACAGCAGTTCTTCTCTGTGG
SMAD7	AAG TCA AGA GGC TGT GTT GCT GT	AGG CTC CAG AAG AAG TTG GGA ATC
SMAD3	GCAGGTGGCTTCCCACATTTGA	TTG CCA AAG CTG GTT ACA GGC CAC
SMAD4	CAACATCCACCAAGTAATCGCGC	AAT TAG GTG TGT ATG GTG CAG TCC TAC T
Collagen 3	ACAGCAGTCCAATGTAGATG	GAGCAGGTGTAGAAGGCTG
Osteopontin	GAC TTT AAG CAA GAA ACT CTT CCA AGC AA	TGT GGC ATC GGG ATA CTG TTC ATC AGA AA
CCN2	ACC TAG AGG AAA ACA TTA AGA AGG GCA AA	CAT ACA TCT TCC TGT AGT ACA AGG ACT CAA AGA T

GAPDH	GCTAACATCAAATGGGGTGATG CTG	GAGATGATGACCCTTTTGGCCCCAC

Table S2. Changes in Echocardiographic parameters in the Sham, Ligated and Ligated+ATE1 knockdown rats

TREATMENT GROUPS	LVDD (mm)	%FS
Sham	4.24	62.08
Sham	4.11	61
Ligated	5.03	34.6
Ligated	4.8	36.2
Ligated	4.95	35.4.3
Ligated+ATE1/CMC-peptide	4.35	52.4
Ligated+ATE1/CMC-peptide	4.22	47.6
Ligated+ATE1/CMC-peptide	4.30	49

Figure Legends:

Supplementary Figure S1: Western blot (uncropped) results of ATE1 protein levels in heart samples from sham and renal ligated rats.

Supplementary Figure S2: Western blot (uncropped) results of ANP in Ligated ATE1 siRNA when compared with Ligated NS SiRNA samples.

Supplementary Figure S3-S7: Representative western blots (uncropped) showing the protein level of P-TAK1, TAK-1, P-JNK1/2 & JNK1/2, P- ERK1/2 (S4J) ERK-1/2 in Ligated ATE1 si RNA and ligated NS siRNA.

Supplementary Figure S8-S9: Representative western blots (uncropped) showing the protein level of Smad3, Smad4 in (Ligated ATE1 siRNA) vs Ligated+ NS siRNA.

Supplementary Figure S10: Representative western blots (uncropped) showing the protein level of osteopontin in the indicated groups.

Supplementary Figure S11: Representative western blots (uncropped) and quantitative analysis showing the protein level of Caspase3 in the indicated groups

Supplementary Figure S12: Cartoon representation of the summary of ATE1 role in regulating cardiac hypertrophy. In the present study, we demonstrated that the cardiac function can be improved by the reduced expression levels of ATE1 and that can be achieved by blocking three proposed signaling pathways. First, activated TAK1 results in activation of JNK1/2 signaling pathway, enhances hypertrophic markers expression, and subsequently leads to cardiac hypertrophy Second, TGFβ-Smad signaling activated due to pressure overload, increase expression of fibrotic markers and result in collagen synthesis. Third, hypertrophic stimuli also activate apoptotic signaling, ultimately leading to apoptosis. ATE1 knockdown blocks the TAK dependent cardiac hypertrophy pathway and TGFβ-Smad signaling dependent cardiac fibrosis. This knockdown also blocks caspase 3 activation.
